# Supplementary material for: mRNA Cap Methylation in Pluripotency and Differentiation
Source: Cell Rep. 2016 Jul 21;16(5):1352–65. doi: 10.1016/j.celrep.2016.06.089 (PMC4977272; doi:10.1016/j.celrep.2016.06.089)
Supplement: Document S1. Supplemental Experimental Procedures and Figures S1–S10 [file mmc1.pdf]

Cell Reports, Volume *16*

Supplemental Information

mRNA Cap Methylation in Pluripotency and Differentiation

Laura Grasso, Olga Suska, Lindsay Davidson, Thomas Gonatopoulos-Pournatzis, Ritchie Williamson, Lize Wasmus, Simone Wiedlich, Mark Peggie, Marios P. Stavridis, and Victoria H. Cowling

Figure S1

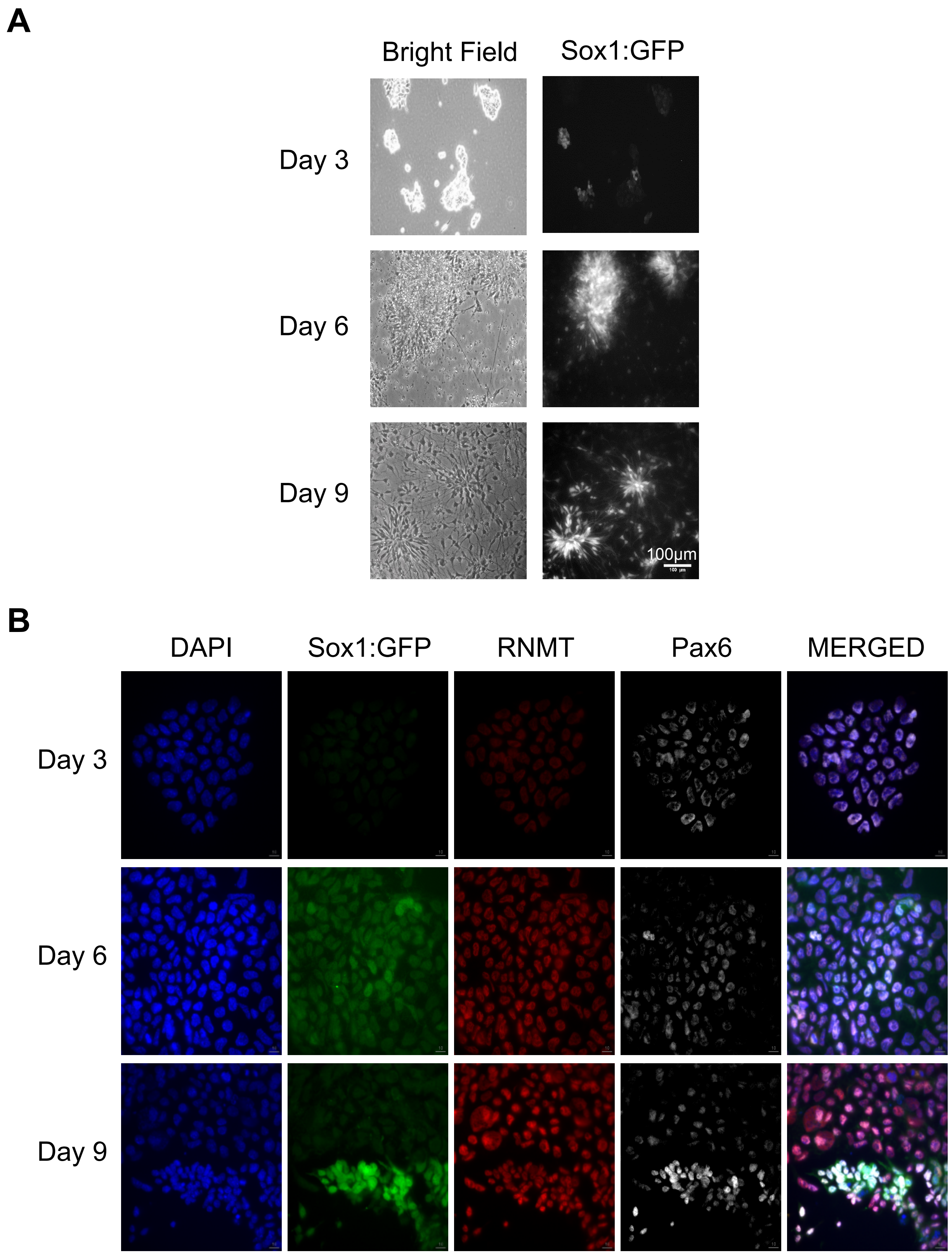

Figure S1 (related to Figure 1) Establishing neural differentiation of ESC
Detection of morphological changes in ES cells during neural differentiation. ESCs were seeded in N2B27 media on dishes coated with 0.1% gelatin. Media was changed every second day. Phase-contrast and flourescent images were captured on days 3, 6 and 9. Scale bar measures 100 µm.
b) Immunofluorescence microscopy was used to detect Sox1-GFP and PAX6, markers of neural differentiation. ESCs were seeded in N2B27 media onto coverslip coated with Laminin. Cells were fixed with 4% PFA and stained with the indicated antibodies on days 3, 6 and 9. DAPI staining was used to detect nuclei. High-resolution images were collected using a DeltaVision Restoration; Applied Precision system, using a 40X/1.514 oil Olympus objective lens. Images were processed using OMERO software.

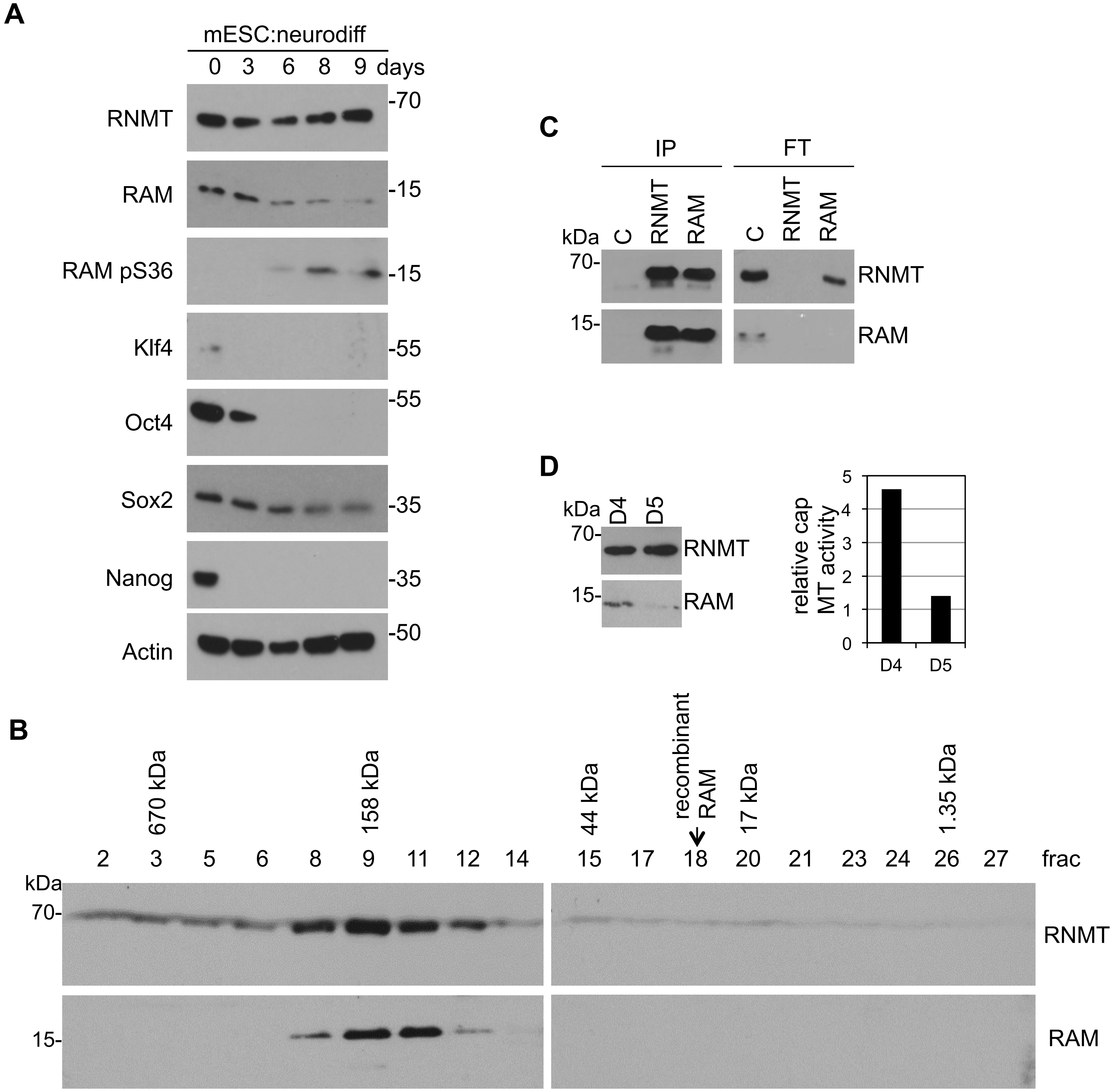

Figure S2 (related to Figure 1) RNMT-RAM in ESCs

a) ESCs were cultured according to a 9-day neural differentiation protocol. Proteins detected by WB. b) 0.5mg ESC extract was separated on a Superdex S200 column in 150 mM NaCl, 50 mM HEPES 0.03 % Brij-35 and 1mM DTT, pH 7.5 and 0.5ml fractions collected. Western blots were performed to detect RNMT and RAM in selected fractions. The migration of markers and recombinant RAM is indicated above blots. c) 10µg ESC extract was subject to immunoprecipitation using anti-GST (c), anti-RNMT and anti-RAM antibodies. Following immuoprecipitation, immunoprecipitates (IP) and residual cell extract (FT) was analysed by western blot for expression of RNMT and RAM. d) The cap methyltransferase activity of ESCs and differentiated cells was analysed by *in vitro* assay. 2µg nuclear extract was incubated with an excess of GpppG-RNA substrate and 100nM S-adenosyl methionine for 7 mins at 30ºC. Products were digested with P1 nuclease and analysed by thin layer chromatography to quantitate m7GpppG-RNA produced. Assays were performed according to (Cowling, 2010).

Figure S3

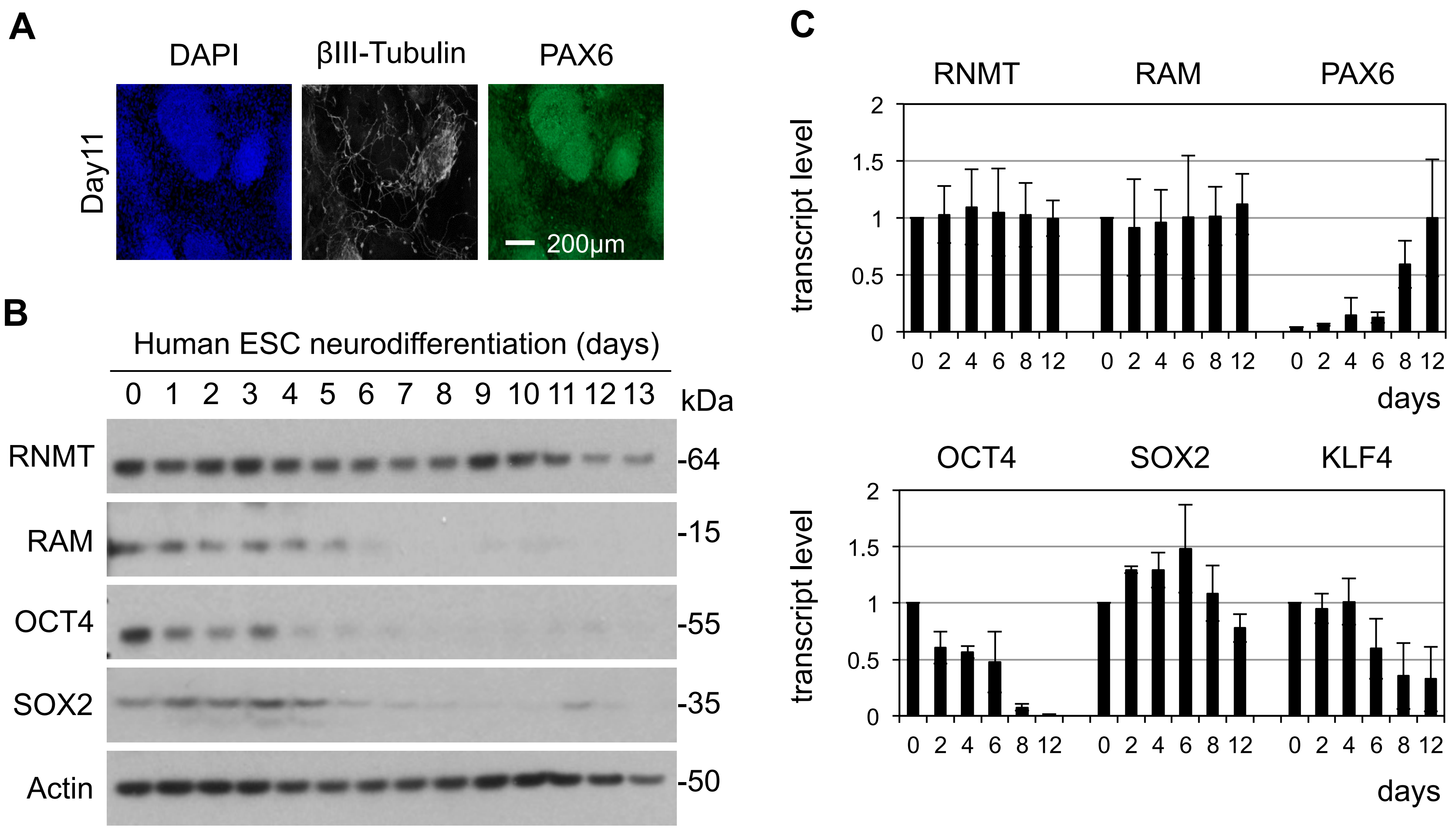

Figure S3 (related to Figure 1) Expression of RAM is suppressed during human ESC neural differentiation
hES cells SA181were subject to neural differentiation protocol. a) Day 11 cultures were analysed for βIII-Tubulin and PAX6 expression by immunofluorescence. b) Cell extracts were analysed by WB. c) Transcript levels were determined by RTPCR relative to Actin expression. Chart represents average and standard deviation of data from three independent experiments.

Figure S4

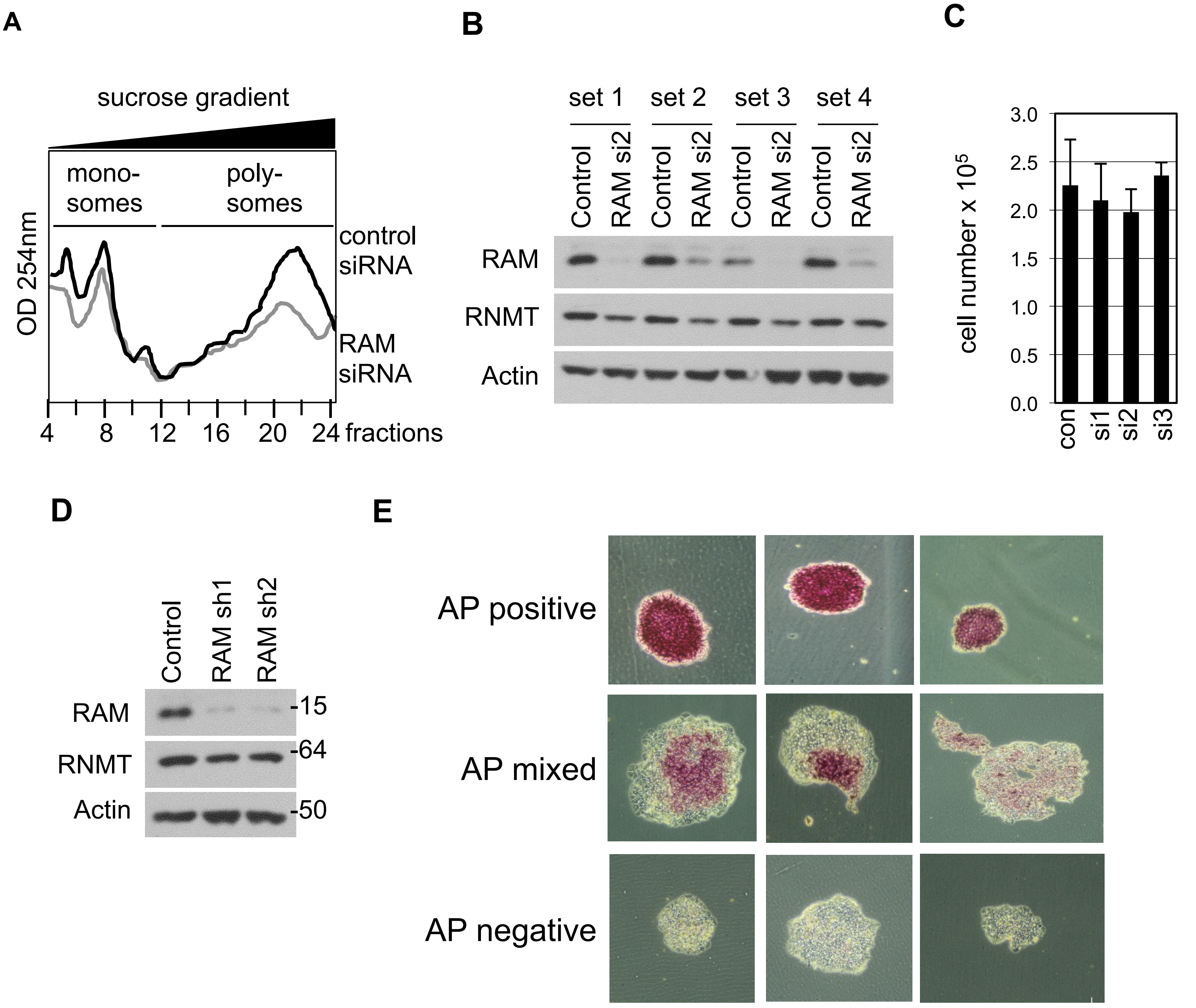

Figure S4 (related to Figure 2) Transfection of ESCs with RAM siRNA and shRNA

ESCs were transfected with a RAM siRNA2 or a non-targetting control. a) After 48hrs RNA-protein complexes were resolved through a sucrose gradient. Protein-RNA complexes detected by absorbance at 254nm. b) ESCs were transfected with RAM siRNA 2 on 4 independent occasssions. After 48hrs RAM protein level was detected. c) 48hrs after transfection with three independent RAM siRNAs cell number was counted. d) Cells were infected with lentiviruses expressing RAM shRNA or control. After 7 days protein levels were detected by western blot. f) Colonies were stained with alkaline phosphatase. Colonies representative of those scored in figure 2d.

Figure S5

Ensembl Gene ID	Gene Name	log FC t'script	P value t'script	control polysome loading	Reference
			**Pluripotency**		
ENSMUSG00000024406	Pou5f1 (Oct4)	-0.188	1.43E-04	1.585	(Chen et al., 2008; Dunn et al., 2014; Kim et al., 2008)
ENSMUSG00000074637	Sox2	-0.092	5.72E-02	1.024	(Boroviak et al., 2015; Chen et al., 2008; Dunn et al., 2014; Kim et al., 200
ENSMUSG00000012396	Nanog	-0.291	3.78E-07	1.619	(Boroviak et al., 2015; Chen et al., 2008; Dunn et al., 2014; Kim et al., 200
ENSMUSG00000003032	Klf4	-0.140	5.35E-03	1.052	(Boroviak et al., 2015; Chen et al., 2008; Dunn et al., 2014; Kim et al., 200
ENSMUSG00000022346	Myc	0.092	2.60E-01	1.184	(Chen et al., 2008; Kim et al., 2008)
ENSMUSG00000037169	Mycn	-0.007	8.94E-01	1.643	(Chen et al., 2008)
ENSMUSG00000004040	Stat3	-0.056	2.66E-01	0.582	(Boroviak et al., 2015; Chen et al., 2008; Dunn et al., 2014)
ENSMUSG00000031681	Smad1	-0.155	2.91E-02	1.320	(Chen et al., 2008)
ENSMUSG00000005698	Ctcf	-0.0865	8.22E-02	0.860	(Chen et al., 2008)
ENSMUSG00000025056	Nr0b1	-0.081	1.10E-01	1.940	(Boroviak et al., 2015; Dunn et al., 2014; Kim et al., 2008)
ENSMUSG00000027490	E2f1	-0.164	1.35E-02	0.158	(Chen et al., 2008)
ENSMUSG00000021255	Esrrb	-0.248	3.53E-06	0.536	(Chen et al., 2008; Dunn et al., 2014)
ENSMUSG00000001910	Nacc1	-0.158	1.38E-03	0.699	(Chen et al., 2008)
ENSMUSG00000055024	Ep300	-0.104	5.44E-02	0.655	(Chen et al., 2008)
ENSMUSG00000051176	Zfp42	-0.111	2.04E-02	1.805	(Kim et al., 2008)
ENSMUSG00000017548	Suz12	-0.055	2.52E-01	0.801	(Chen et al., 2008)
ENSMUSG00000026380	Tfcp2l1	-0.246	9.94E-05	0.264	(Boroviak et al., 2015; Chen et al., 2008; Dunn et al., 2014)
ENSMUSG00000041483	Zfp281	-0.020	6.87E-01	0.540	(Kim et al., 2008)
ENSMUSG00000079509	Zfx	-0.207	5.03E-04	0.694	(Chen et al., 2008)
ENSMUSG00000055148	Klf2	0.046	5.23E-01	2.079	(Boroviak et al., 2015; Dunn et al., 2014)
ENSMUSG00000018604	Tbx3	-0.338	7.38E-08	0.747	(Dunn et al., 2014)
ENSMUSG00000034486	Gbx2	-0.082	1.98E-01	1.438	(Dunn et al., 2014)
ENSMUSG00000027547	Sall4	0.028	5.58E-01	0.971	(Dunn et al., 2014)
ENSMUSG00000020167	Tcf3	-0.079	1.26E-01	1.114	(Boroviak et al., 2015; Dunn et al., 2014)
ENSMUSG00000005148	Klf5	-0.060	2.23E-01	1.141	(Boroviak et al., 2015; Dunn et al., 2014)
ENSMUSG00000035478	Mbd3	-0.042	4.70E-01	1.170	(Dunn et al., 2014)
ENSMUSG00000051176	Zfp42	-0.111	2.04E-02	1.805	(Boroviak et al., 2015; Dunn et al., 2014)
ENSMUSG00000030353	Tead4	-0.125	7.10E-02	0.394	(Boroviak et al., 2015)
ENSMUSG00000026398	Nr5a2	-0.197	4.16E-04	0.854	(Boroviak et al., 2015)
ENSMUSG00000072419	Dppa2	0.111	3.08E-02	1.735	(Boroviak et al., 2015)
ENSMUSG00000020594	Pum2	-0.115	1.76E-02	0.854	(Boroviak et al., 2015)
ENSMUSG00000032446	Eomes	-0.317	1.24E-03	1.430	(Boroviak et al., 2015)
ENSMUSG00000042414	Prdm14	-0.517	3.93E-06	0.926	(Boroviak et al., 2015)
ENSMUSG00000032494	Tdgf1	-0.183	8.17E-04	1.506	(Boroviak et al., 2015)
ENSMUSG00000021835	Bmp4	-0.130	3.05E-02	1.039	(Boroviak et al., 2015)
ENSMUSG00000030117	Gdf3	-0.018	7.01E-01	2.476	(Boroviak et al., 2015)
ENSMUSG00000000730	Dnmt3l	0.558	3.07E-12	1.887	(Boroviak et al., 2015)
ENSMUSG00000025856	Pdgfa	-0.128	2.93E-02	0.667	(Boroviak et al., 2015)
ENSMUSG00000055799	Tcf7l1	-0.009	9.29E-01	1.630	(Boroviak et al., 2015)
ENSMUSG00000047751	Utf1	-0.213	9.06E-05	2.130	(Boroviak et al., 2015)
ENSMUSG00000067261	Foxd3	-0.277	7.35E-05	1.655	(Boroviak et al., 2015)
ENSMUSG00000067860	Zic3	-0.296	1.77E-05	1.019	(Boroviak et al., 2015)
ENSMUSG00000023902	Zscan10	-0.0085	8.69E-01	0.688	(Boroviak et al., 2015)
ENSMUSG00000050917	Fgf4	-0.419	4.24E-10	0.763	(Boroviak et al., 2015)
ENSMUSG00000090125	Pou3f1	-0.466	1.48E-03	0.725	(Boroviak et al., 2015)
ENSMUSG00000021848	Otx2	-0.238	1.72E-02	0.856	(Boroviak et al., 2015)
			**Chromating remodelling**		
ENSMUSG00000030034	Ino80b	-0.61	1.08E-06	2.13	(Wang et al., 2014)
ENSMUSG00000047989	Ino80c	-0.26	1.67E-04	0.66	(Wang et al., 2014)
ENSMUSG00000030689	Ino80e	-0.13	1.50E-02	1.02	(Wang et al., 2014)
ENSMUSG00000040865	Ino80d	-0.14	1.05E-02	0.14	(Wang et al., 2014)
ENSMUSG00000030689	Ino80e	-0.13	1.50E-02	1.02	(Wang et al., 2014)
			**RNA PolII related**		
ENSMUSG00000042487	Leo1	-0.01	9.01E-01	1.65	(Ponnusamy et al., 2009)
ENSMUSG00000045106	Ccdc73	0.07	4.97E-01	0.88	(Ponnusamy et al., 2009)
ENSMUSG00000005609	Ctr9	0.04	4.55E-01	0.60	(Ponnusamy et al., 2009)
ENSMUSG00000027304	Rtf1	-0.18	4.71E-03	1.20	(Ponnusamy et al., 2009)
ENSMUSG00000003437	Paf1	0.19	2.52E-04	1.05	(Ponnusamy et al., 2009)
ENSMUSG00000043866	Taf10	0.01	8.86E-01	0.73	(Pijnappel et al., 2013)
ENSMUSG00000024218	Taf11	-0.02	8.06E-01	1.13	(Pijnappel et al., 2013)
ENSMUSG00000048100	Taf13	-0.04	6.05E-01	0.54	(Pijnappel et al., 2013)
ENSMUSG00000025049	Taf5	-0.24	3.87E-03	1.38	(Pijnappel et al., 2013)
ENSMUSG00000028899	Taf12	-0.20	5.88E-03	0.84	(Pijnappel et al., 2013)
ENSMUSG00000078941	Ak6	-0.27	3.50E-03	1.12	(Pijnappel et al., 2013)

Figure S5 (related to Figure 3) List of pluripotency-associated factors
Transcript logFC in siRAM/Ct and polysome loading in ESCs of genes previously implicated in embryonic stem cell function. Median polysome loading is 0.99. For full references see main text.

Figure S6

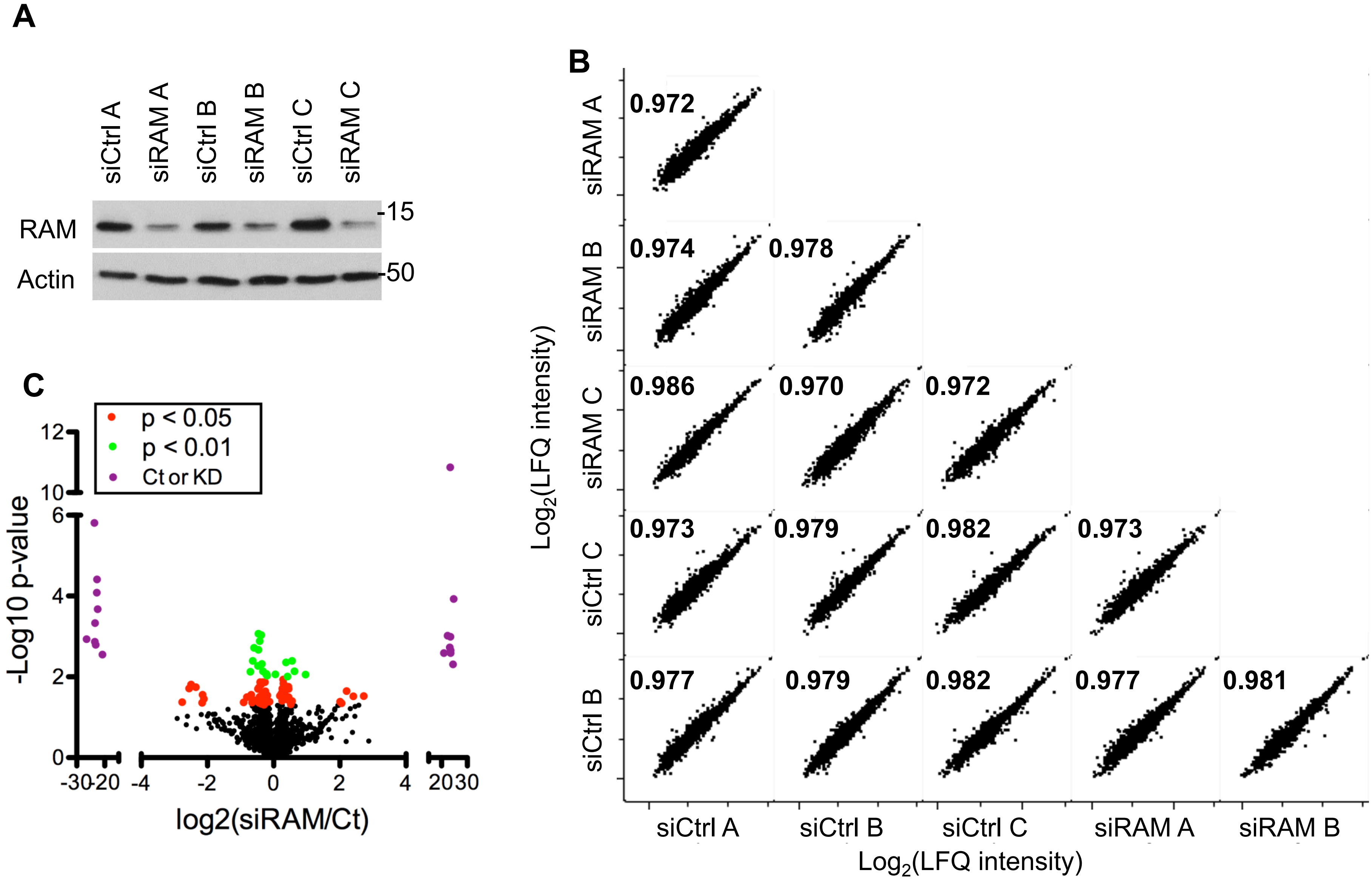

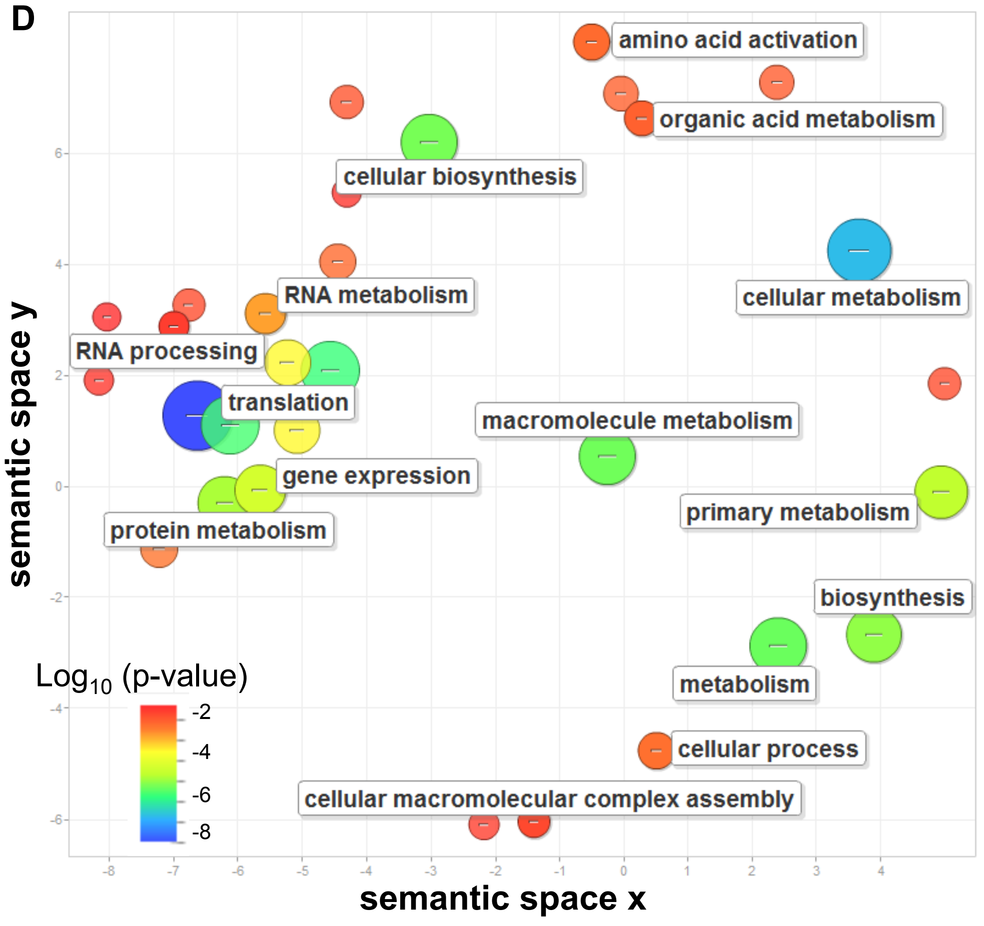

Figure S6 (related to Figure 3) LC-MS/MS analysis of the global proteome in response to RAM repression

ESCs were transfected with RAM siRNA pool or non-targeting control for 48hrs. a)Western blot analysis performed to detect proteins indicated. b) Samples analysed by LC-MS/MS, proteins identified and label –free quantified (LFQ) by MaxQuant (http://141.61.102.17/maxquant_doku/) and a scatter plot matrix for all biological replicates generated using Perseus framework (http://141.61.102.17/perseus_doku). Numbers indicate Pearson correlation between indicated samples. c) Cellular proteins quantified using LC-MS/MS. Each spot represents one of 2627 proteins analysed for differential expression. The y-axis plots the P-value (-log10 transformed) derived from Student's T-test across three biological replicates. The x-axis plots the mean log2 ratio of expression in RAM knock-down samples to control. The utmost left- and right-hand site spots (purple) represent proteins detected only in control or RAM knockdown samples respectively. d) The list of downregulated proteins after RAM knock-down (p value ≤ 0.05) analysed for the enrichment of GO biological process terms using DAVID database. Results rationalised using REVIGO. The colour and size of each bubble represent enrichment p-value (EASE score). The proximity of bubbles reflects related GO terms.

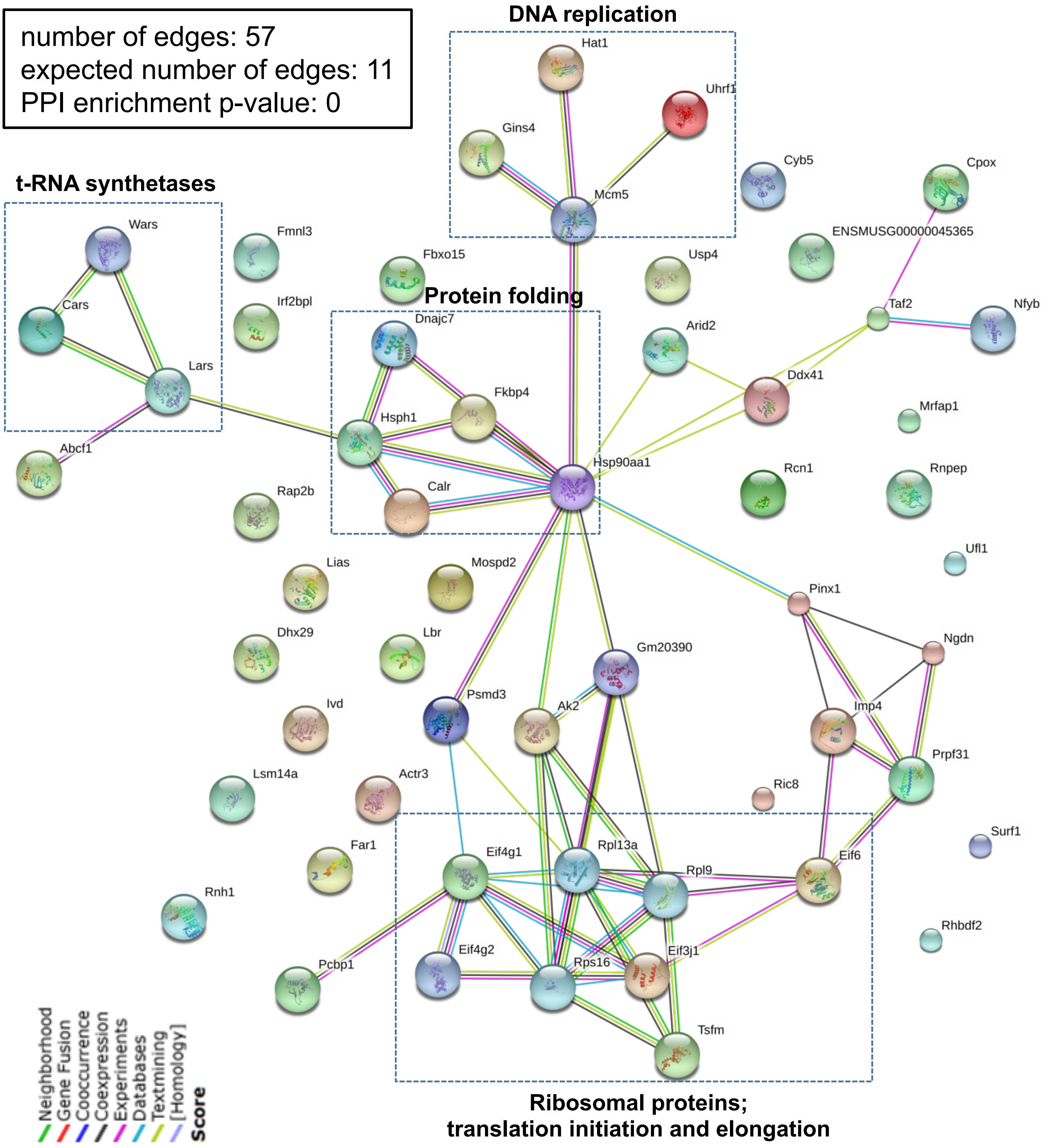

Figure S7 (related to Figure 3) STRING analysis of proteins downregulated after RAM knockdown
The list of downregulated proteins after RAM knockdown (p value ≤ 0.05) was analysed using STRING: functional protein association networks database (http://string-db.org/). Proteins were clustered based on the STRING Evidence Score. Gene clusters of interest are indicated.

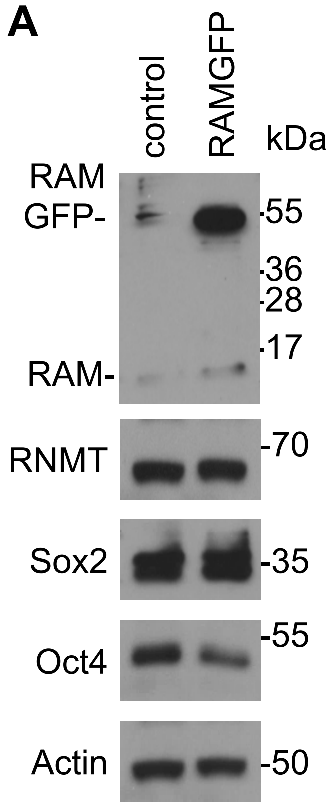

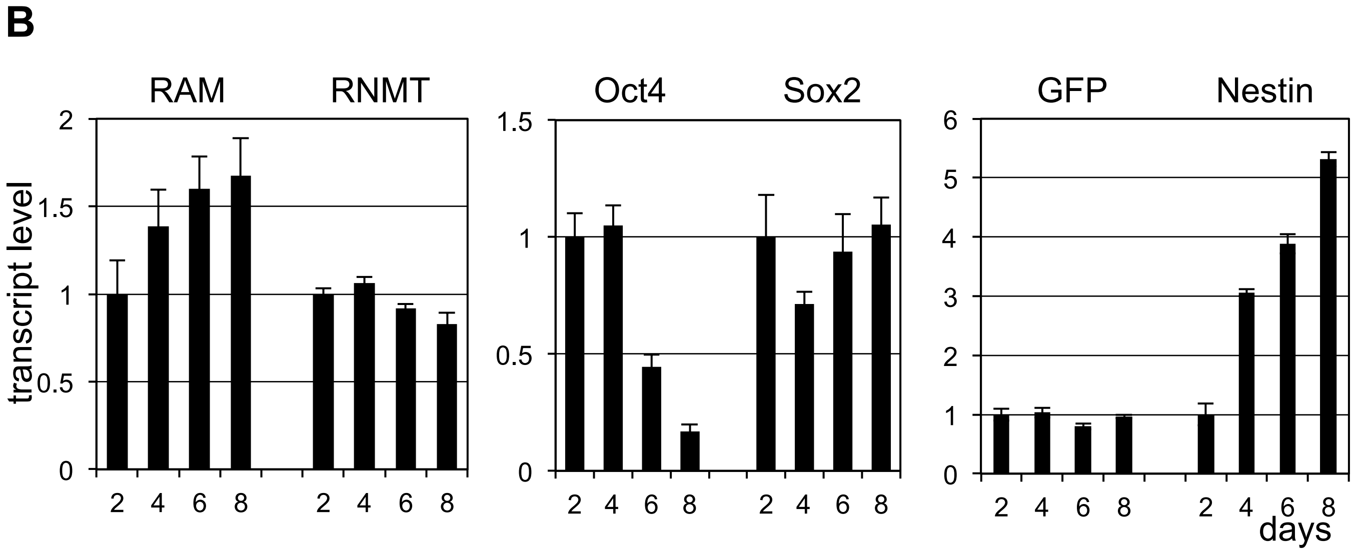

Figure S8 (related to Figure 4) Differentiation of ESC: RAM-GFP

a) In ESC:RAM-GFP and ESC:control, expression of the proteins indicated was detected by Western blot. b) ESCs stably expressing RAM-GFP were cultured according to a 9-day neural differentiation protocol. RT-PCR was performed for the genes indicated, expressed relative to Actin transcripts.

Figure S9

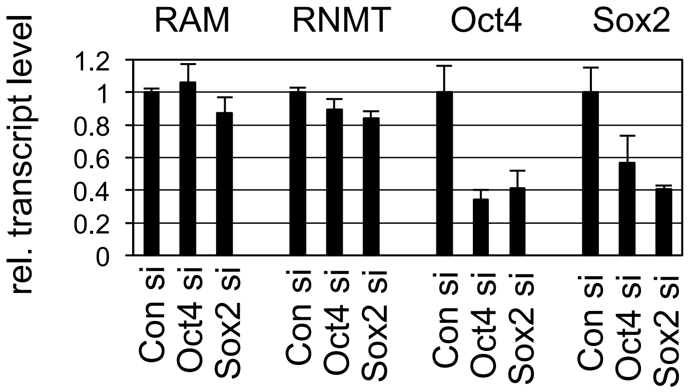

Figure S9 (related to Figure 5) RNMT and RAM transcript levels are not reduced by repression of Oct4 or Sox2
Expression of Oct4 and Sox2 was inhibited by transfection of siRNA pools for 72hrs. RTPCR performed to detect transcript expression relative to Actin transcripts.

Figure S10

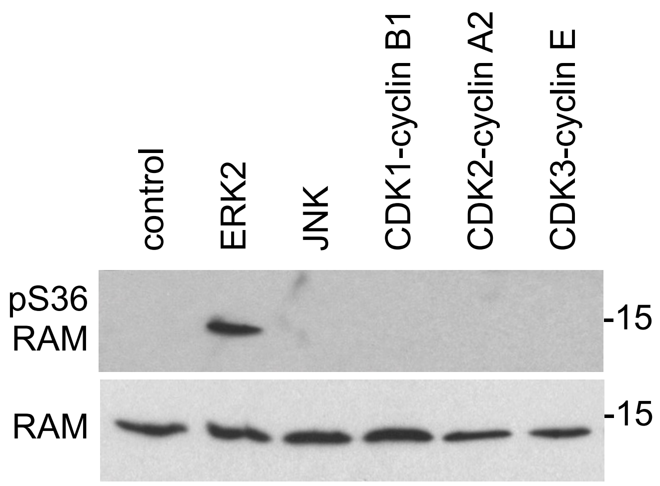

Figure S10 (related to Figure 6) ERK2 phosphorylates RAM
10ng GST-RAM subject to kinase reaction with kinases indicated was analysed by WB for pS36 RAM and total RAM.

Supplementary experimental procedures

ShRNA usage

For shRNA stable transduction non-targeting or RAM shRNA coding plasmids (MISSION Sigma) were co-transfected with envelope and packaging plasmids into HEK293T cells. The supernatant containing lentivirus was used to transduce mESCs and cells were selected with 1μg/ml Puromycin.

Alkaline Phosphatase

For clonal analysis, 200-600 cells were plated in the well of a 6-well plate and after 7 days fixed in citrate-acetone-formaldehyde and stained using the Alkaline Phosphatase (AP) kit (Sigma – Aldrich) according to the manufacturer's instructions. Colonies were scored for each experimental well as AP positive (cells all AP positive), mixed (containing cells both AP positive and negative) and or negative (cells all negative) (Figure S4E)

Polysome profile

Cells were incubated in 100 μg/ml cycloheximide for 10 min, washed in ice cold PBS supplemented with 100 μg/ml cycloheximide and extracts were collected in polysome lysis buffer: 15 mM Tris (pH7.5), 15 mM MgCl2, 0.3 M NaCl, 1 mM DTT, 1% Triton X-100, 100 mg/ml cycloheximide, 100 U/ml RNasin. 10% of extracts was kept as input and the rest layered onto 10 ml of 10%–50% sucrose steps and centrifuged at 18 000 x g for 2 hr at 4ºC. 0.5 ml fractions were collected and OD_{254} nm monitored. RNA was extracted from fractions using RNeasy Mini Kit (QIAGEN) according to manufacturer's instructions.

RTPCR

RNA was extracted using Trizol Reagent (Invitrogen). cDNA was synthesised using iScript cDNA Synthesis Kit (Bio-Rad). qRT-PCR was performed using QuantaBiosciences SYBR Green FastMix for iQ.

m7G IP

dT-purified RNA was IPed with 10 μl anti-7-methyl guanosine (Cole and Cowling, 2009). Transcripts were analyzed by real-time PCR and IPs expressed relative to inputs.

RNA-sequencing and analysis

Fractions 17-22, assessed to be polysomal, were purified by Phenol:Chloroform:Isoamyl Alcohol (25:24:1) (Ambion), pooled, and RNA precipitated with 2M of LiCl (Sigma). RNA from inputs was purified using Trizol Reagent (Invitrogen) according to manufacturer's instructions. RNA was sequenced at the Tayside Centre for Genomic Analysis. RNA was quality controlled using TapeStation (Agilent Technologies). RNA sequencing libraries were prepared with the TruSeq Stranded Total RNA with Ribo-Zero kit (Illumina). Sequencing was performed using NextSeq Series High Output Kit 2 × 75 bp (Illumina).

Reads were quality controlled using FastQC, mapped to (GRCm38/mm10) assembly of the mouse genome using STAR 2.5.1b (Dobin et al., 2013) and number of reads per gene quantified using htseq-count tool (Anders et al., 2015). Differentially expressed genes were identified using edgeR package (Robinson et al., 2010). Genes that had at least 1 counts per million (CPM) in at least 10 samples (inputs or polysomes) were analysed for differential expression. Polysome loading was calculated as ratio of gene abundance in polysome fraction over input in control sample. Median polysome loading 0.99.

Gene set tests were performed using ROAST which calculates the direction of changes of a gene set taking into account the magnitude of expression changes in the experiment (Wu et al., 2010). Gene sets tested were: pluripotency-associated factors (for full list see Fig S6), genes belonging to ESC expression group (upregulated in ESCs) or neurogenic progenitors (tNPs and nNPs) expression group (upregulated during N2B27 neural differentiation)(Abranches et al., 2009) (Table S1), the PluriNet protein-protein network shared by the pluripotent cells (Muller et al., 2008)(downloaded from Molecular Signatures Database, MSigDB http://software.broadinstitute.org/gsea/msigdb), genes upregulated in 6 human embryonic stem cell lines (Bhattacharya et al., 2004)(from MSigDB), genes coordinately upregulated in a compendium of mouse ESCs, which are shared with the human ESC-like module (Wong et al., 2008)(from MSigDB), genes involved in Neuronal System from Reactome Pathway Database (http://www.reactome.org)(from MSigDB), neuron markers - genes enriched in neurons in the adult mouse brain identified through correlation-based searches seeded with neuron cell-type specific gene expression patterns (Lein et al., 2007)(from MSigDB).

Proteomics

Cells were lysed in 8 M Urea, 100 mM Tris, pH 8.0 lysis buffer supplemented with 2x Complete protease inhibitor cocktail tablets (Roche) and 1x PhosSTOP phosphatase inhibitor cocktail tablets (Roche). 300 μg protein/sample was reduced with 10 mM DTT (Formedium) for 30 min

and thiol groups were alkylated with 50 mM iodoacetamide for 45 min. Samples were diluted to 0.8 M Urea and proteins digested with Trypsin Gold (Promega) two times. Samples were desalted on SepPak C18 SPE cartridges (Waters), washing with 0.1% trifluoroacetic acid (TFA) (Thermo), and eluted with 50% Acetonitrile (ACN) (VWR)/0.1%TFA. Eluted samples were dried, resuspended in 10 mM borate buffer, pH 9.3, 20% ACN and separated into 10 fractions on Dionex IonPac AS24 Hydroxide-Selective Anion-Exchange Column. Fractions were desalted as previously on Sep Pak tC18 elution plate, dried and resuspended in of 5% formic acid (Sigma). Peptides (0.7 µg/fraction) were analysed using Ultimate 3000 RSLCnano system (Thermo Scientific) coupled to a Q Exactive HF OrbiTrap Mass Spectrometer (Thermo Scientific). The tandem mass spectrometry analysis was carried out using HCD fragmentation. The data dependent acquisition mode was used to acquire MS/MS spectra from the top 20 most abundant ions from the full scan MS. The RAW data was processed using MaxQuant software (version 1.5.0.0) (Cox and Mann, 2008) using Label-free quantification (LFQ) mode. MS/MS spectra were searched against mouse UniProt database (17/03/15) using the Andromeda search engine. Peptide and protein level identification were both set to maximum false discovery rate of 1%. For protein quantification match between runs function was enabled and minimum ratio count was set to two. Protein groups identified as contaminants or reverse sequences were removed. Statistical analysis was done in Microsoft Excel. P values were calculated using *Student's t test.* For calculation of log2 ratio in RAM knock-down samples to control values 0 were replaced with 1. The Gene Ontology (GO) analysis was performed using DAVID Functional Annotation Tool (Huang da et al., 2009). Significantly enriched GO biological process terms (*modified Fisher Exact P-Value* – EASE score ≤ *0.05)* were visualised using REVIGO (Supek et al., 2011). GO term enrichment analysis was performed using STRINGdb Bioconductor package (Franceschini et al., 2013).

Western Blotting

Cells were lysed in lysis buffer (10 mM Tris (pH 7.05), 50 mM NaCl, 50 mM NaF, 10% glycerol, 0.5% Triton X-100 and protease inhibitors). Cell extracts were normalised for protein content and typically 8-20 ug was analysed by Western Blot. Primary antibodies used were raised against RNMT (polyclonal sheep), RAM (polyclonal sheep), pS36 RAM (polyclonal sheep), ERK 1/2 (Cell Signalling 9102) pERK 1/2 (Cell Signalling 9101S), Oct4 (Abcam 18976), Sox2 (Cell Signaling 4900), Nanog (Abcam 80892), Klf4 (Santa Cruz 20691), Actin (Abcam 3280.) FG-M2 (Sigma F1804) and GFP (Roche 11814460001), Ubiquitin (Dako Z0458).

Immunofluorescence

ES cells were seeded onto cover glass previously coated with 0.1% gelatin whereas neurons were seeded onto cover glass coated with Laminin (Sigma) for 2 hr. Cells were fixed with PFA 4% for 30 min, permabilised with 1% Triton X-100 in PBS and then blocked with 1% Donkey serum in PBS with 0.05% Tween 20 for 30 min. Cells were then incubated for 1 hr with primary antibodies RNMT (1:500, in house), RAM (1:100, in house), Oct4 (1:500, Abcam) Pax 6 (1:500, Santa Cruz) and beta-III-tubulin (Sigma T5076, 1:1000). Cells were then washed three times with PBS to be then incubated for 1 hr with secondary antibodies (Alexa) in the dark. Nuclei were stained with DAPI (Sigma).

hES cells were washed twice with PBS, fixed for 10 min with 10% neutral buffered formalin (Sigma). Cells were then washed with PBS and treated with ice cold 90% methanol for 5 min at -20°C. Cells were then blocked for 1 hr with 4% BSA in PBS/0.1 % Triton X-100 (blocking buffer), then treated as above. Antibodies used: beta-III-tubulin (Sigma T5076, 1:1000) and Pax6 (Abcam 154253, 1:200).

High-resolution images were collected with an imaging system (DeltaVision Restoration; Applied Precision) using a 40X/ 1.514 oil or 60X/1.42 oil (Olympus) objective lens. Images were then processed using OMERO software.

Quantification of the fluorescence intensity of Oct4, Nanog and Sox2 in cells transfected with three independent siRNAs or a non-targeting control for 48 hours. Measurements were performed using the freeware image analysis software, Image J. Data indicate the mean ± standard deviation from at least ten images from two independent experiments.

Kinase assay

500ng GST-RAM was incubated with 100ng kinase at 37ºC for 30 minutes in 50mM Tris pH 7.5, 0.1% B-mercaptoethanol, 10mM Mg Acetate, 100μM ATP and 1μM Microcystin LR. Kinases used were ERK2 (DSTT), CDK2-cyclin A2 and JNK (DSTT), CDK1-cyclin B1 (NEB) and CDK3-cyclin E (Thermo Scientific). Kinases were verified as active by DSTT (Aregger et al., 2016)

RAM-GFP expression

RAM-GFP was cloned into the vector pPyPCAGIP (Chambers et al., 2003). In this vector RAM-GFP is driven by the CAG chimeric enhancer/promoter consisting of a CMV early enhancer, chicken beta-actin promoter, first exon and first intron and the splice acceptor from rabbit beta

globin (Niwa et al., 1991). RAM is expressed upstream of IRES-Puro allowing puromycin selection. This promoter is resistant to silencing in ESCs.

References

Abranches, E., Silva, M., Pradier, L., Schulz, H., Hummel, O., Henrique, D., and Bekman, E. (2009). Neural differentiation of embryonic stem cells in vitro: a road map to neurogenesis in the embryo. PloS one *4*, e6286.
Anders, S., Pyl, P.T., and Huber, W. (2015). HTSeq--a Python framework to work with high-throughput sequencing data. Bioinformatics (Oxford, England) *31*, 166-169.
Aregger, M., Kaskar, A., Varshney, D., Fernandez-Sanchez, M.E., Inesta-Vaquera, F.A., Weidlich, S., and Cowling, V.H. (2016). CDK1-Cyclin B1 Activates RNMT, Coordinating mRNA Cap Methylation with G1 Phase Transcription. Mol Cell *61*, 734-746.
Bhattacharya, B., Miura, T., Brandenberger, R., Mejido, J., Luo, Y., Yang, A.X., Joshi, B.H., Ginis, I., Thies, R.S., Amit, M.*, et al.* (2004). Gene expression in human embryonic stem cell lines: unique molecular signature. Blood *103*, 2956-2964.
Chambers, I., Colby, D., Robertson, M., Nichols, J., Lee, S., Tweedie, S., and Smith, A. (2003). Functional expression cloning of Nanog, a pluripotency sustaining factor in embryonic stem cells. Cell *113*, 643-655.
Cole, M.D., and Cowling, V.H. (2009). Specific regulation of mRNA cap methylation by the c-Myc and E2F1 transcription factors. Oncogene *28*, 1169-1175.
Cowling, V.H. (2010). Enhanced mRNA cap methylation increases cyclin D1 expression and promotes cell transformation. Oncogene *29*, 930-936.
Cox, J., and Mann, M. (2008). MaxQuant enables high peptide identification rates, individualized p.p.b.-range mass accuracies and proteome-wide protein quantification. Nature biotechnology *26*, 1367-1372.
Dobin, A., Davis, C.A., Schlesinger, F., Drenkow, J., Zaleski, C., Jha, S., Batut, P., Chaisson, M., and Gingeras, T.R. (2013). STAR: ultrafast universal RNA-seq aligner. Bioinformatics (Oxford, England) *29*, 15-21.
Franceschini, A., Szklarczyk, D., Frankild, S., Kuhn, M., Simonovic, M., Roth, A., Lin, J., Minguez, P., Bork, P., von Mering, C.*, et al.* (2013). STRING v9.1: protein-protein interaction networks, with increased coverage and integration. Nucleic Acids Res *41*, D808-815.
Huang da, W., Sherman, B.T., and Lempicki, R.A. (2009). Systematic and integrative analysis of large gene lists using DAVID bioinformatics resources. Nat Protoc *4*, 44-57.
Lein, E.S., Hawrylycz, M.J., Ao, N., Ayres, M., Bensinger, A., Bernard, A., Boe, A.F., Boguski, M.S., Brockway, K.S., Byrnes, E.J.*, et al.* (2007). Genome-wide atlas of gene expression in the adult mouse brain. Nature *445*, 168-176.
Muller, F.J., Laurent, L.C., Kostka, D., Ulitsky, I., Williams, R., Lu, C., Park, I.H., Rao, M.S., Shamir, R., Schwartz, P.H.*, et al.* (2008). Regulatory networks define phenotypic classes of human stem cell lines. Nature *455*, 401-405.
Niwa, H., Yamamura, K., and Miyazaki, J. (1991). Efficient selection for high-expression transfectants with a novel eukaryotic vector. Gene *108*, 193-199.
Robinson, M.D., McCarthy, D.J., and Smyth, G.K. (2010). edgeR: a Bioconductor package for differential expression analysis of digital gene expression data. Bioinformatics (Oxford, England) *26*, 139-140.
Supek, F., Bosnjak, M., Skunca, N., and Smuc, T. (2011). REVIGO summarizes and visualizes long lists of gene ontology terms. PloS one *6*, e21800.
Wong, D.J., Liu, H., Ridky, T.W., Cassarino, D., Segal, E., and Chang, H.Y. (2008). Module map of stem cell genes guides creation of epithelial cancer stem cells. Cell Stem Cell *2*, 333-344.

Wu, D., Lim, E., Vaillant, F., Asselin-Labat, M.L., Visvader, J.E., and Smyth, G.K. (2010). ROAST: rotation gene set tests for complex microarray experiments. Bioinformatics (Oxford, England) *26*, 2176-2182.
