# Supplementary material for: mRNA Cap Methylation in Pluripotency and Differentiation
Source: Cell Rep. 2016 Jul 21;16(5):1352–65. doi: 10.1016/j.celrep.2016.06.089 (PMC4977272; doi:10.1016/j.celrep.2016.06.089)
Supplement: Document S2. Article plus Supplemental Information [file mmc7.pdf]

Cell Reports

Article

mRNA Cap Methylation in Pluripotency and Differentiation

Graphical Abstract

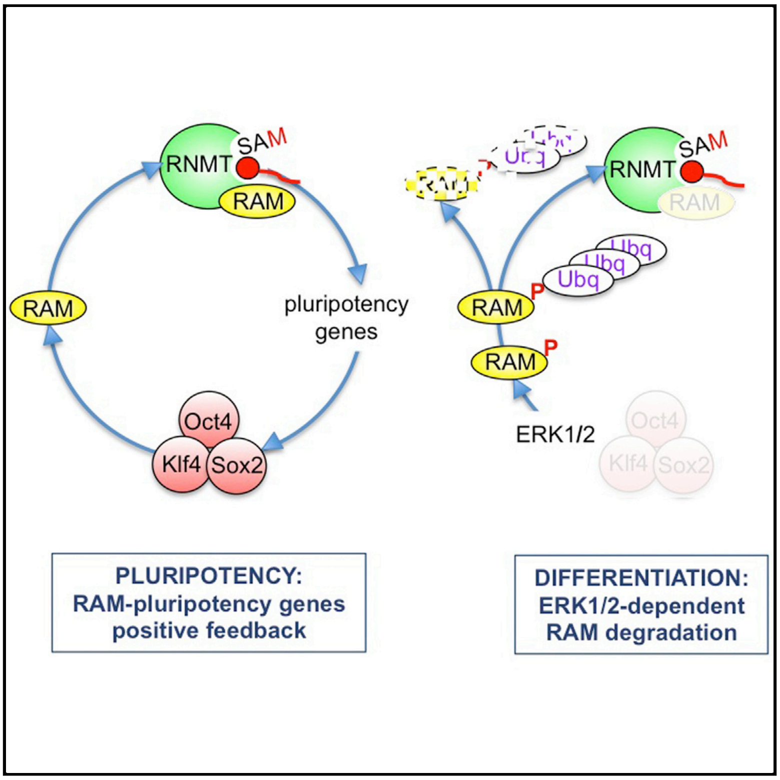

Highlights

- The mRNA cap methyltransferase RNMT-RAM is highly expressed in embryonic stem cells
- RNMT-RAM is important for the expression of pluripotency-associated genes
- During neural differentiation, the cap methyltransferase activator RAM is repressed
- Repression of RAM contributes to upregulation of neural genes and neural morphology

Authors

Laura Grasso, Olga Suska,
Lindsay Davidson, ..., Mark Peggie,
Marios P. Stavridis, Victoria H. Cowling

Correspondence

v.h.cowling@dundee.ac.uk

In Brief

The mRNA cap stabilizes transcripts and recruits processing and translation factors. Grasso et al. report that the mRNA cap methyltransferase RNMT-RAM is highly expressed in embryonic stem cells and is important for pluripotency-associated gene expression. Repression of RAM occurs during neural differentiation and is important for expression of neural-associated genes.

Accession Numbers

GSE81849

Grasso et al., 2016, Cell Reports *16*, 1352–1365
August 2, 2016 © 2016 The Authors.
http://dx.doi.org/10.1016/j.celrep.2016.06.089

OPEN ACCESS
CellPress

Cell Reports
Article

mRNA Cap Methylation in Pluripotency and Differentiation

Laura Grasso,[1,8] Olga Suska,[1,8] Lindsay Davidson,[2] Thomas Gonatopoulos-Pournatzis,[6] Ritchie Williamson,[7] Lize Wasmus,[3] Simone Wiedlich,[4] Mark Peggie,[4] Marios P. Stavridis,[5] and Victoria H. Cowling[1,3,*]
[1]Centre for Gene Regulation and Expression
[2]Human Pluripotent Stem Cell Facility
[3]MRC Protein Phosphorylation and Ubiquitylation Unit
[4]Department of Signal Transduction Therapies
[5]Division of Cell and Developmental Biology
School of Life Sciences, University of Dundee, Dow Street, Dundee DD1 5EH, UK
[6]Donnelly Centre, University of Toronto, Toronto, ON M5S 3E1, Canada
[7]School of Pharmacy, Faculty of Life Sciences, University of Bradford, Bradford, West Yorkshire BD7 1DP, UK
[8]Co-first author
*Correspondence: v.h.cowling@dundee.ac.uk
http://dx.doi.org/10.1016/j.celrep.2016.06.089

SUMMARY

The mRNA cap recruits factors essential for transcript processing and translation initiation. We report that regulated mRNA cap methylation is a feature of embryonic stem cell (ESC) differentiation. Expression of the mRNA cap methyltransferase activating subunit RAM is elevated in ESCs, resulting in high levels of mRNA cap methylation and expression of a cohort of pluripotency-associated genes. During neural differentiation, RAM is suppressed, resulting in repression of pluripotency-associated factors and expression of a cohort of neural-associated genes. An established requirement of differentiation is increased ERK1/2 activity, which suppresses pluripotency-associated genes. During differentiation, ERK1/2 phosphorylates RAM serine-36, targeting it for ubiquitination and proteasomal degradation, ultimately resulting in changes in gene expression associated with loss of pluripotency. Elevated RAM expression also increases the efficiency of fibroblast reprogramming. Thus, the mRNA cap emerges as a dynamic mark that instructs change in gene expression profiles during differentiation and reprogramming.

INTRODUCTION

In pluripotent stem cells, differential expression of a network of transcription factors governs the selection of self-renewal or differentiation (Dunn et al., 2014; Tanabe et al., 2014). A stable pluripotent state can be induced in somatic cells by expression of four transcription factors: Oct4, Sox2, Klf4, and c-Myc (Takahashi et al., 2007; Takahashi and Yamanaka, 2006). Chromatin modifiers and remodelers and the transcriptional machinery facilitate the function of the core transcription factors during differentiation and reprogramming (Bickmore and Zaret, 2010). However, changes in transcription alone cannot achieve the proteomic requirements of pluripotency and differentiation. Notably, an initial study that generated induced pluripotent stem cells required enhanced expression of LIN28, which promotes translation by antagonizing Let7 microRNA (miRNA) function (Yu et al., 2007). Influential RNA regulons that drive changes in the translational profile during differentiation and reprogramming can be coordinated by RNA modifications, RNA binding proteins, miRNAs, or the translation machinery (Jia et al., 2012; Stunnenberg et al., 2015; Tahmasebi et al., 2014; Wang et al., 2013; Wong et al., 2016).

During transcription, pre-mRNA receives a series of modifications to create the 5′ mRNA cap that protects nascent transcripts from exonucleases and forms a recruitment platform for cap-binding complexes, which mediate gene expression mechanisms, including RNA splicing, nuclear export, and translation initiation (Gonatopoulos-Pournatzis and Cowling, 2014a; Shuman, 2015; Topisirovic et al., 2011). The basic mRNA cap structure (cap 0) is 7-methylguanosine linked via triphosphate to the first transcribed nucleotide (X), m7G(5′)ppp(5′)X. Cap formation is catalyzed by a series of enzymes (Topisirovic et al., 2011). In mammals, RNGTT has triphosphatase and guanylyltransferase activities that add the guanosine cap to the nascent transcript. Subsequently, the guanosine cap is methylated on the N-7 position by RNMT-RAM, creating the mature cap 0 structure. RNMT-RAM is critical for gene expression, since the completion of cap 0 renders transcripts competent to be processed and translated.

The mammalian cap methyltransferase consists of a catalytic subunit, RNMT (RNA guanine-7 methyltransferase), and an activating subunit, RAM (RNMT-activating miniprotein) (Gonatopoulos-Pournatzis et al., 2011; Topisirovic et al., 2011). RNMT has basal methyltransferase activity, which RAM increases 5- to 10-fold. RNMT and RAM also stabilize each other and have only been isolated as a complex. RAM consists an N-terminal

 © 2016 The Authors.
This is an open access article under the CC BY license (http://creativecommons.org/licenses/by/4.0/).

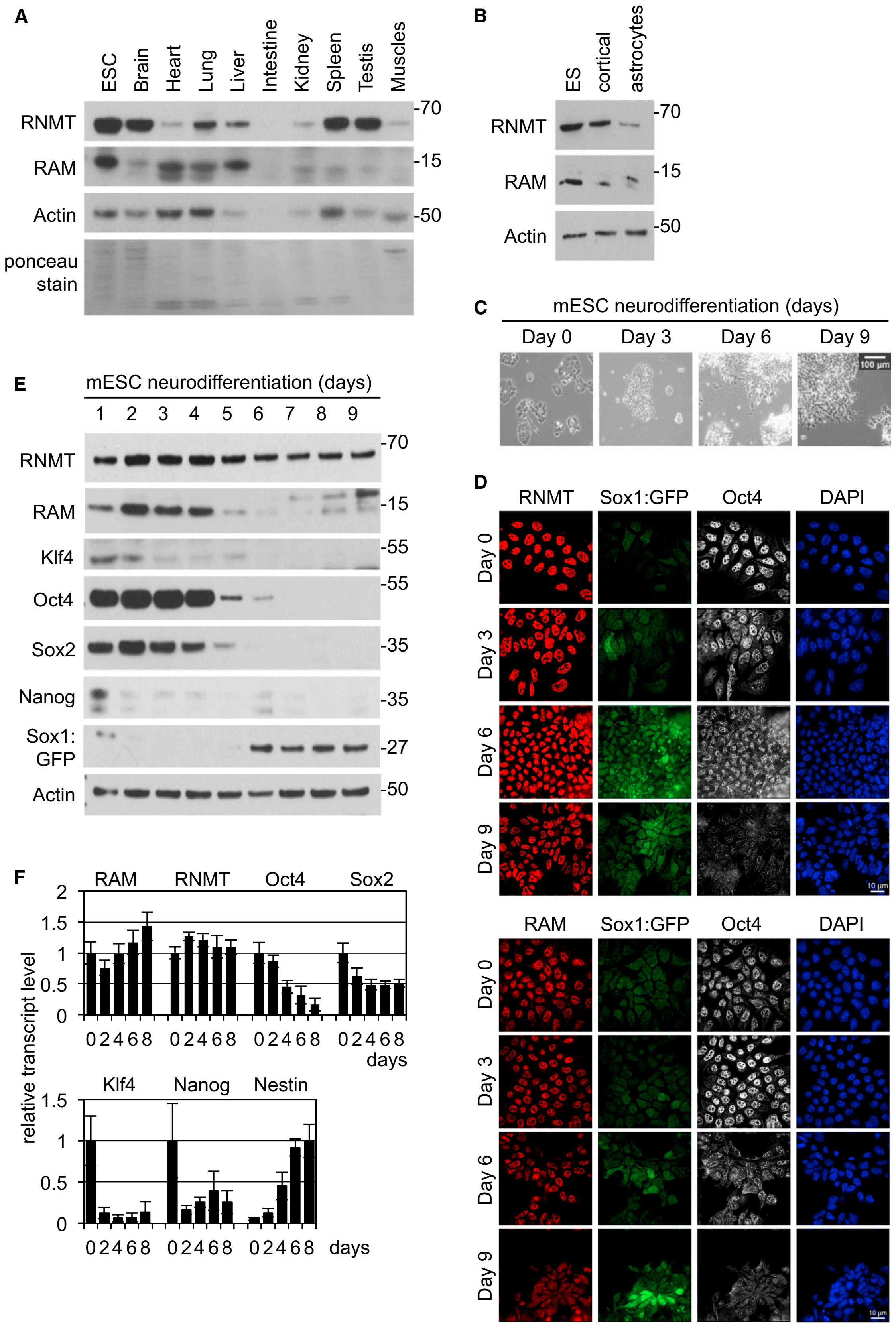

(legend on next page)

activation domain, a central RNA binding domain that increases RNA recruitment, and a C-terminal nuclear localization domain (Gonatopoulos-Pournatzis and Cowling, 2014b). RNMT-RAM is recruited to phosphorylated RNA pol II, coordinating capping with the initiation of transcription (Aregger and Cowling, 2013; Glover-Cutter et al., 2008). Both RNMT and RAM expression was found to be required for gene expression, cell proliferation, and viability (Chu and Shatkin, 2008; Gonatopoulos-Pournatzis et al., 2011).

Formation of the mRNA cap is incomplete on certain transcripts and regulated by cellular signaling pathways (Cole and Cowling, 2009; Cowling and Cole, 2007; Fernandez-Sanchez et al., 2009; Jiao et al., 2010, 2013; Mukherjee et al., 2012). In yeast, incomplete mRNA caps are associated with RNA degradation, whereas in mammals, transcripts with incomplete caps can be stable but translated at a vastly reduced rate (Cowling and Cole, 2007; Fernandez-Sanchez et al., 2009; Mukherjee et al., 2012). CDK1 phosphorylates and activates RNMT, coordinating G1 transcription with mRNA cap methylation (Aregger et al., 2016). The transcription factors c-Myc and E2F-1 upregulate RNA pol II phosphorylation, which increases RNMT-RAM recruitment to transcription initiation sites and stimulates mRNA cap formation (Aregger and Cowling, 2012; Cole and Cowling, 2009; Cowling and Cole, 2007). c-Myc-dependent mRNA cap methylation is also dependent on upregulation of SAHH, the enzyme that hydrolyses the inhibitory byproduct of methylation (Fernandez-Sanchez et al., 2009).

Here, we report that RNMT-RAM is regulated during embryonic stem cell (ESC) differentiation and fibroblast reprogramming. Expression of a cohort of pluripotency-associated genes is dependent on high levels of the cap methyltransferase activator RAM, present in embryonic or induced pluripotent stem cells. During differentiation, ERK1/2-dependent phosphorylation triggers RAM degradation, resulting in repression of pluripotency-associated genes and expression of differentiation-associated genes.

RESULTS

RNMT and RAM Are Differentially Expressed in Primary Tissues

The expression of the mRNA cap methyltransferase RNMT and its activator, RAM, was investigated in a panel of mouse organs and ESCs (Figure 1A). RNMT and RAM expression varied across the panel, with the highest expression of RNMT in brain, spleen, and testis and the highest expression of RAM in heart, lung, and liver. At extremes, brain tissue had significant RNMT expression but minimal RAM expression, whereas heart tissue had minimal RNMT expression but significant RAM expression. For this analysis, polyclonal antibodies raised against full-length RNMT and RAM that recognize epitopes across the proteins were used, and therefore, loss of signal is likely due to loss of protein and not post-translational modification or specific isoform expression (Aregger and Cowling, 2013; Gonatopoulos-Pournatzis and Cowling, 2014b). The observations made with the organ panel imply that different cell lineages will contain different RNMT to RAM ratios. Since brain tissue exhibited a high RNMT to RAM ratio, these proteins were analyzed in primary neural cells. In murine primary cortical neurons, the RNMT level was equivalent to that in ESCs, whereas the RAM level was reduced (Figure 1B). In astrocytes, RNMT and RAM expression was minimal compared to ESCs.

RAM Expression Is Reduced during Neural Differentiation

Since RAM activates RNMT, reduced RAM expression was predicted to have consequences for mRNA cap formation and gene expression. We utilized an ESC differentiation protocol to investigate RAM function during neural differentiation (Figures 1C–1F, S1A, S1B, and S2A) (Wongpaiboonwattana and Stavridis, 2015; Ying et al., 2003). During this protocol, the emergence of neural morphology; loss of ESC pluripotency factors Oct4, Klf4, Sox2, and Nanog; and gain of neural markers Nestin, Sox1 (observed by GFP Sox1 locus knockin, $Sox1^{Gt(EGFP)Asmi}$), and Pax6 confirmed differentiation into neural precursors (Aubert et al., 2003). During differentiation, the reduction in Klf4 and Nanog protein levels reflected changes in transcript levels, implying predominantly transcriptional control of these genes (Figures 1E, 1F, and S2A). However, Oct4 and Sox2 protein levels were maintained from day 1 to 5/6 of differentiation, despite transcript levels falling significantly during this period (Figures 1E and 1F). This implies significant post-transcriptional control of Oct4 and Sox2 during the initial days of differentiation, maintenance of Oct4 and Sox2 translation rates, and/or inhibition of protein degradation.

During neural differentiation, RNMT protein levels decreased slightly, whereas RAM protein levels decreased significantly by day 5 (Figures 1E and S2A), resulting in a high RNMT to RAM ratio, which was also observed in brain tissue and cortical neurons (Figures 1A and 1B). RAM transcript levels were maintained during neural differentiation, and therefore, RAM protein is reduced by a post-transcriptional mechanism (Figure 1F). Similar reductions in RNMT and RAM expression were observed during differentiation of human SA181 ESCs into neural precursors (Figure S3) (Chambers et al., 2009). Loss of SOX2 and OCT4 expression, gain of PAX6 and βIII-tubulin, and the appearance of neurites indicated loss of pluripotency and induction of the neural phenotype (Figures S3A–S3C). During differentiation,

Figure 1. Expression of RAM Is Suppressed during Murine Neural Differentiation
(A) Mouse organ and murine ESC extracts analyzed by western blot (WB). Ponceau S was used to stain PVDF membranes.
(B) ESCs, primary cortical neurons, and primary astrocytes extracts analyzed by WB. ESCs were cultured according to a 9-day neural differentiation protocol.
(C) Phase-contrast images of cultures. Scale bar, 100 μm.
(D) Proteins, including Sox1 (GFP reporter), were detected by immunofluorescence (IF). Scale bar, 10 μM. DAPI DNA stain was used.
(E) Proteins detected by WB.
(F) Transcripts detected by RT-PCR, normalized to Actin. Data represent an average of three independent experiments, and error bars indicate SD.
See also Figures S1–S3.

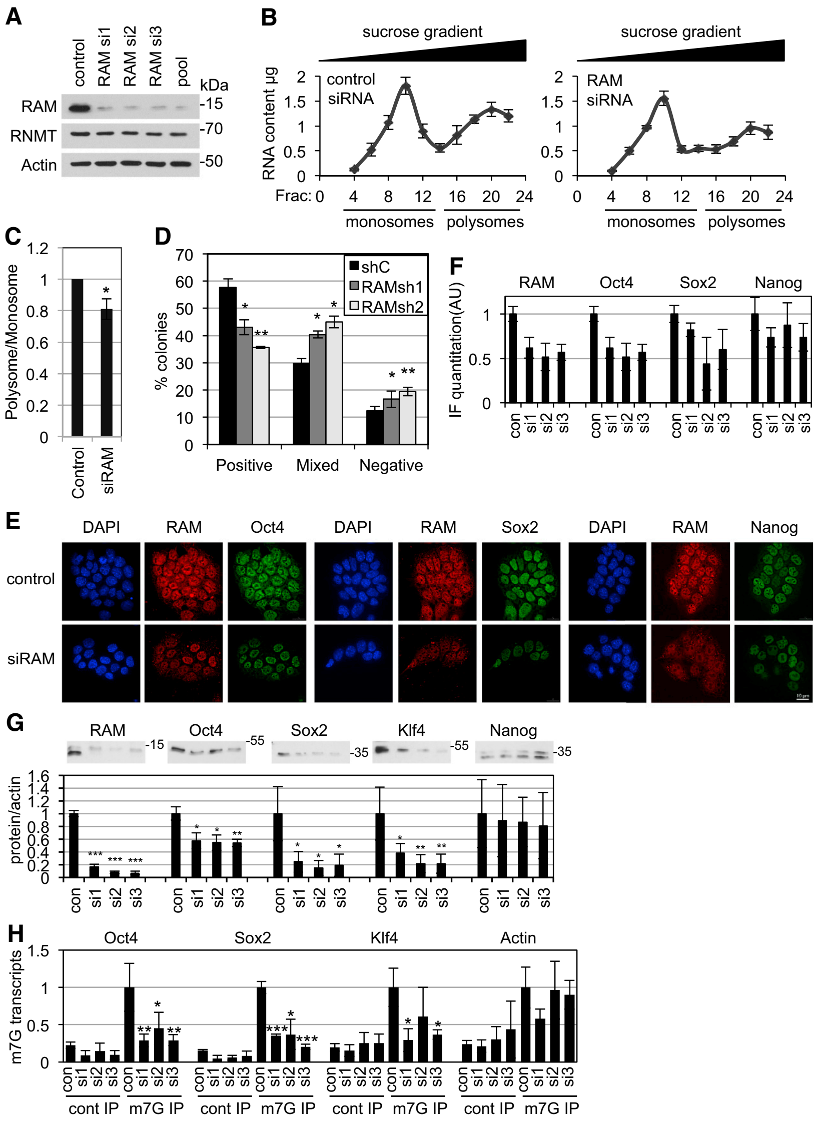

Figure 2. RAM Is Important for Oct4, Sox2, and Klf4 Expression

(A) ESCs transfected with three independent siRNAs, pool, or a non-targeting control for 48 hr.

(B) RNA-protein complexes resolved through sucrose gradient. RNA was purified and quantitated. Mean and SEM of four independent experiments are presented.

(C) Relative ratio of monosomes to polysomes.

(D) ESCs infected with lentiviruses carrying RAM or control small hairpin RNAs (shRNAs), seeded at clonal density, and stained for alkaline phosphatase (AP) after 7 days. Over 290 colonies were scored in three independent experiments, and average percent AP positive, mixed, or negative colonies are reported.

(E) ESCs were transfected with RAM or control siRNA for 48 hr and fixed, and proteins were detected by IF. Scale bar, 10 µm.

(F) Quantitation of average cellular staining in five independent fields (ImageJ software). Data are representative of two independent experiments.

(G) For at least three independent experiments, average protein level relative to actin was detected by WB and quantitated.

(H) Anti-m7G antibodies or matched controls used to immunoprecipitate m7G-RNA (RT-PCR analysis).

(C, D, G, and H) Error bars indicate SD for at least three independent experiments. t test p value relative to control (*$p < 0.05$; **$p < 0.01$, ***$p < 0.001$).

See also Figure S4.

RNMT expression reduced slightly whereas RAM expression was minimal by day 7 (Figure S3B). As in murine ESC differentiation, a reduction in RAM transcripts did not accompany loss of RAM protein, which is therefore repressed by a post-transcriptional mechanism (Figure S3C).

In order to understand the consequences of reduced RAM expression, it was important to determine whether in ESCs RAM functions only in a complex with RNMT or also functions as a monomer or in other complexes. In previously investigated cell lines, RAM was only observed in a complex with RNMT (Gonatopoulos-Pournatzis et al., 2011). In ESCs, RAM monomers were not detected by gel filtration (Figure S2B). Using reciprocal immunoprecipitations (IPs), RNMT and RAM were found in a complex (Figure S2C). RNMT IP significantly depleted RAM from cell extracts, whereas RAM IP only partially depleted RNMT, consistent with the majority of RAM being complexed with RNMT. A likely scenario is that during differentiation, RNMT-RAM complexes decrease and RNMT monomers increase. Consistent with a reduction in RAM, day 5 neural precursors exhibited reduced cap methyltransferase activity compared to day 4 (Figure S2D).

RAM Is Important for mRNA Translation and Oct4, Sox2, and Klf4 Expression

We investigated whether the high RAM levels in ESCs have biological significance. RAM expression was reduced by small interfering RNA (siRNA) transfection, reproducing the minimal expression observed in neural cells (Figure 2A). Since the mRNA cap and mRNA cap methyltransferase can affect mRNA transcription, stability, processing, export, and translation initiation, the effect of RAM reduction on the net output of these events, the translational profile, was investigated. Equivalent numbers of ESCs were plated and transfected with RAM siRNA or control, and 48 hr, later native RNA-protein complexes were separated by centrifugation through a sucrose gradient and detected by UV absorbance (Figure S4A). The experiment was performed on four independent occasions, and the RNA content of fractions was quantitated (Figures 2B and S4B). Free ribosomes and ribosomal subunits (monosomes) were separated from translating RNA-ribosome complexes (polysomes). Reduced RAM expression resulted in a significant reduction in the ratio of polysomes to monosomes, indicating reduced ribosome loading of mRNA and a translation defect (Figure 2C). Note that transfection of RAM siRNA in this experimental protocol resulted in a small reduction in cell number after 2 days

(14.3%, 4.3%SD), accounting for the reduced monosome peak in RAM siRNA-treated cells (Figure S4C).

We investigated the biological impact of RAM on ESCs. Alkaline phosphatase staining can be used as a marker of pluripotency (Martí et al., 2013). On plating ESCs at clonal density, inhibition of RAM resulted in a significant decrease in alkaline phosphatase-positive colonies and an increase in mixed and negative colonies (Figures 2D, S4D, and S4E). Immunofluorescence (IF) analysis indicated that suppression of RAM resulted in reduced expression of the pluripotency-associated transcription factors Sox2 and Oct4, whereas Nanog expression was minimally repressed (Figures 2E and 2F). Western blot analysis of cell extracts confirmed significant repression of Sox2 and Oct4, but not Nanog, protein expression in multiple independent experiments (Figure 2G). Another pluripotency-associated transcription factor, Klf4, was also significantly repressed in response to RAM suppression.

Oct4, Sox2, and Klf4 may be directly or indirectly dependent on RAM for expression. We confirmed that reduced RAM expression results in loss of cap methylation of Oct4, Sox2, and Klf4 transcripts (Figure 2H). The proportion of transcripts with an m7G cap is detected by a semiquantitative method in which m7G-RNA is subject to IP and transcripts detected by RT-PCR (Nanog was not amenable to this technique, potentially due to secondary structure) (Cole and Cowling, 2009). These data indicate that Oct4, Sox2, and Klf4 are likely to be direct targets of RNMT-RAM-dependent cap methylation. However, the dependency of transcripts on the m7G-cap for expression, processing, and translation is gene specific. Therefore, we performed an unbiased analysis of RAM-dependent genes, investigating transcript expression, polysome loading, and protein expression.

RAM Regulates the Expression of Transcripts Associated with Pluripotency

In order to comprehensively identify RAM-dependent genes, we performed a RNA-sequencing (RNA-seq) transcriptome analysis of ESCs transfected with RAM siRNA or control in four biological replicates (Figure S4B). In response to RAM suppression, from 12,803 genes that passed quality thresholds, 2,398 genes were downregulated and 2,569 upregulated (p value < 0.05) (Figure 3A; Table S1). A series of unbiased analyses were performed to determine whether these genes have related biological functions. Gene Ontology (GO) analysis of RAM-regulated transcripts determined that genes associated with developmental processes were among the most significantly enriched (Figure 3B). Gene set enrichment analysis revealed that RAM suppression resulted in significant repression of a set of genes previously implicated in ESC function (p value = 0.002) (Boroviak et al., 2015; Chen et al., 2008; Dunn et al., 2014; Kim et al., 2008) (Figures 3C and S5). Suppression of components of the TFIID and Paf1 complexes, which mediate pluripotency-associated gene expression (Pijnappel et al., 2013; Ponnusamy et al., 2009), and the INO80 complex, which maintains open chromatin at pluripotency gene promoters, were also RAM regulated (Wang et al., 2014) (Figures 3C and S5). Statistically significant repression of Oct4, Klf4, and Nanog transcripts was observed.

Since RAM is reduced during neural differentiation, the contribution of RAM to gene expression changes concomitant with this process was investigated. Previously established ESC and neural-specific gene sets were analyzed (see Supplemental Experimental Procedures). On RAM suppression, all established ESC-specific gene sets tested were significantly downregulated, and all established neuronal-specific gene sets tested were significantly upregulated, including genes activated during N2B27 neural differentiation (Figures 3D and 3E). Thus, high levels of RAM in ESCs are important for the expression of pluripotency-associated genes, and its repression contributes to the upregulation of neural-specific genes.

In order to ascertain the specificity that RAM imparts on RNA translation, the transcript content of polysomes was analyzed in response to RAM suppression (Figure 2B). Out of 12,803 genes analyzed, only 26 genes exhibited RAM-dependent changes in polysome loading normalized to input transcripts (Figure 3F; Table S1). Therefore, RAM does not exhibit specificity over translational control and globally promotes ribosome loading.

Since suppression of RAM results in differential changes in transcript level and global changes in polysome loading, we investigated the resultant effect on the cellular proteome. A proteomic analysis of ESCs transfected with RAM siRNA in three biological replicates was performed using liquid chromatography-tandem mass spectrometry (LC-MS/MS) (Figures S6A and S6B). Proteins were identified and quantified using Maxquant software (Cox and Mann, 2008) (Table S2). Global proteomics studies have the caveat of identifying the most abundant and usually the most stable proteins; however, from the 2,617 proteins that passed quality thresholds, 59 were downregulated and 38 upregulated (p value FDR ≤ 0.05) (Figure S6C; Table S3). GO term analysis revealed that downregulated genes were enriched in categories including "gene expression," "RNA processing," and "translation" (Table S4; Figure S6D). Of note, the translation factors EIF4G1, EIF4G2, EIF3J1, and EIF6; ribosomal proteins RPL13a, RPL9, and RPS16; and tRNA synthetases WARS, CARS, and LARS were all found to be RAM regulated, which is likely to contribute to the global defects in translation (Figure S7). The group of proteins significantly repressed in response to RAM suppression was significantly repressed at the transcript levels, indicating transcript-level control of these genes (p value = 0.01) (Figure 3G; Table S5).

Repression of RAM Is Important for Neural Differentiation

In ESCs, high levels of RAM are important for the expression of pluripotency-associated genes and suppression of RAM induces neural-associated genes. In order to determine whether the repression of RAM observed during neural differentiation contributes to this process, ESCs were engineered to constitutively express RAM-GFP from a plasmid under the control of a chimeric promoter (Figures 4A, 4B, and S8A). These cells were morphologically indistinct from control ESCs (Figure 4A). During neural differentiation, RAM-GFP and endogenous RAM expression was maintained (Figure 4B). RAM-GFP-expressing cells exhibited some features associated with neural differentiation (Figure 4A) and increased Nestin transcripts (Figure S8B).

During neural differentiation of ESC:RAM-GFP, the decrease in Klf4 protein expression was equivalent to that observed in ESCs (Figure 4B). However, expression of the pluripotency markers

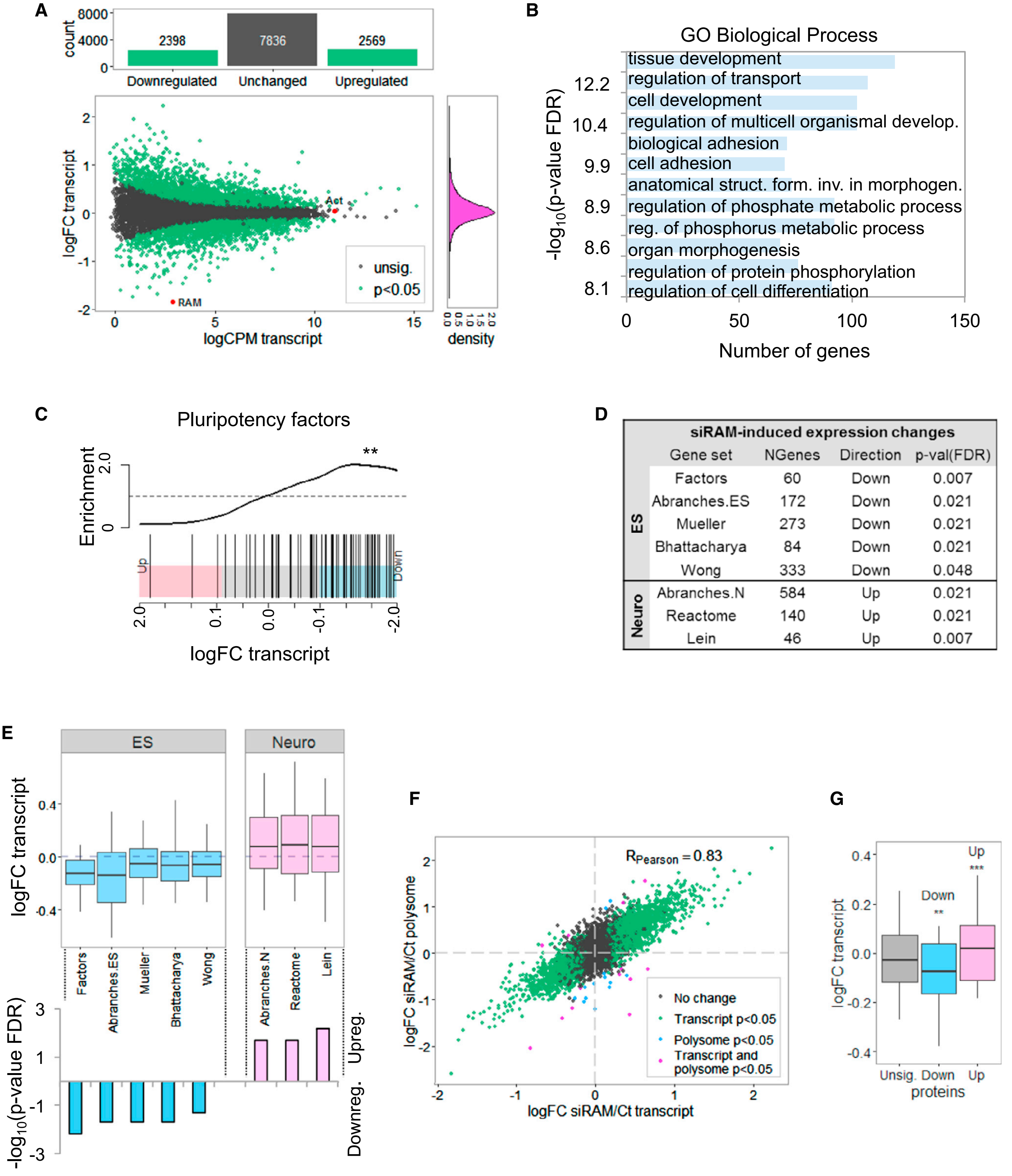

Figure 3. RAM Regulates a Subset of Pluripotency-Associated Genes

(A) Summary of siRAM/control RNA-seq analysis. Number of up- and downregulated genes, scatterplot of transcript log2 fold change (logFC) against transcript abundance (log2 counts per million [logCPM]), and data distribution are shown. RAM and Actin are marked. $p < 0.05$ represents corrected p value < 0.05.

(B) Most enriched GO Biological Process terms for genes up- or downregulated at least 0.5 logFC.

(C) RAM siRNA-induced transcript changes of pluripotency-associated factors (barcode plot). See also Figure S5.

(legend continued on next page)

OPEN ACCESS
CellPress

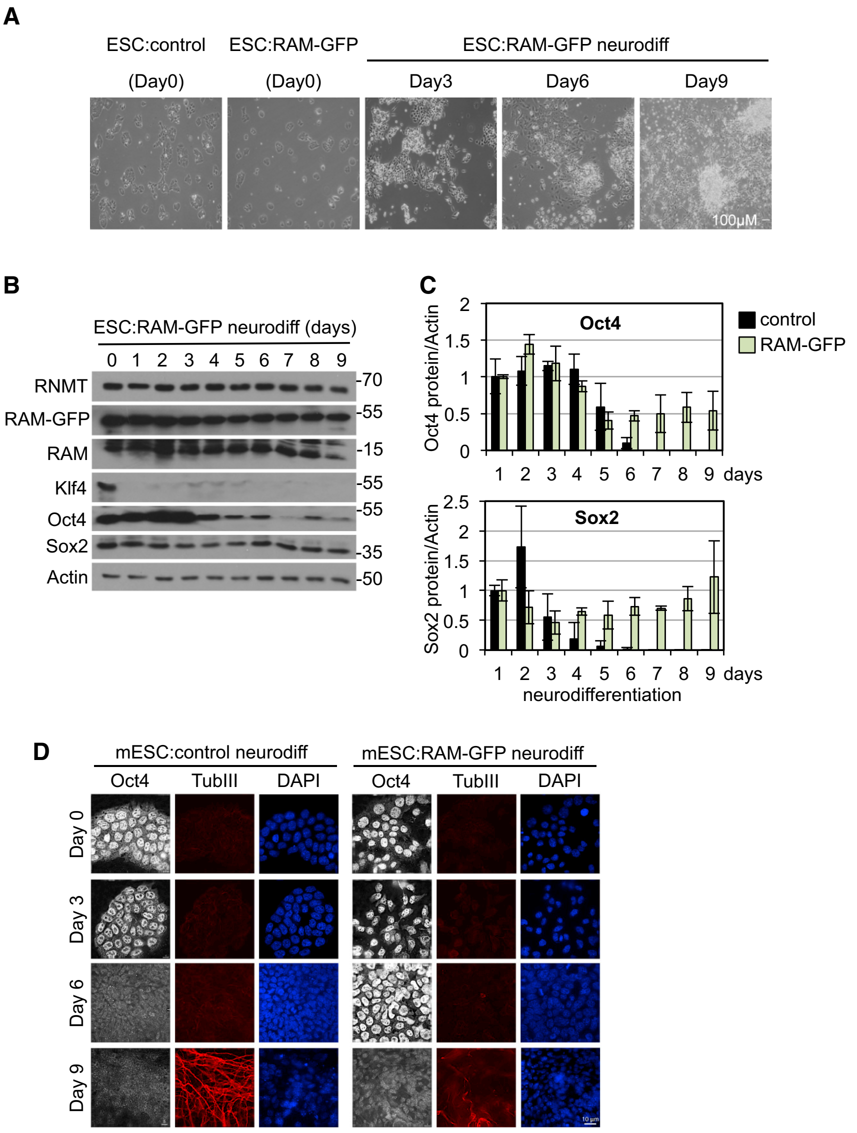

Figure 4. Sustained RAM Expression Maintains Pluripotency Markers during Differentiation

(A) ESC:RAM-GFP or control cultured in a 9-day neural differentiation protocol. Phase-contrast images are shown. Scale bar, 100 μm.

(B) Protein detected by WB.

(C) Oct4 and Sox2 protein level quantitated in three independent experiments. Mean and SD are presented.

(D) Oct4 and tubulin III detected by IF.

See also Figure S8.

Oct4 and Sox2 was partially maintained in ESC:RAM-GFP during differentiation, whereas in control cells, their expression is repressed by day 6 (Figures 1, 4B, and S2A). Quantitation of four independent experiments revealed that Oct4 and Sox2 expression decreases during the initial days of differentiation; however, later during differentiation (days 5–9), Oct4 and Sox2 expression plateaus (Figure 4C). IF analysis indicated that preventing RAM repression delayed and impaired differentiation (Figure 4D). During differentiation of control ESCs, Oct4 expression was minimal by day 6, and expression of the neural marker tubulin III was observed in neurites on day 9. In ESC:RAM-GFP, Oct4 expression was distinct on day 6 and detectable on day 9, and tubulin III was visible on day 9 but less extensive compared to that seen in control cells.

RAM Expression Is Dependent on Oct4 and Sox2

Since RAM controls gene expression in ESCs and during neural differentiation, we investigated the mechanisms that regulate its expression. Initial investigation focused on the pluripotency-associated transcription factors Oct4, Sox2, Klf4, and Nanog (Figures 5A–5C). Inhibition of Oct4 and Sox2 expression by single or siRNA pool transfection resulted in a reduction in RAM protein expression, whereas inhibition of Klf4 and Nanog did not. Consistent with previous studies, transfection of Oct4 or Sox2 siRNA reduced expression of Klf4 and Nanog (Figure 5A) (Dunn et al., 2014). It is possible that RAM is repressed in response to severe disruption of this transcriptional network rather than specifically in response to Oct4 and Sox2 inhibition. Regulation of RAM expression did not appear directly transcriptional; suppression of Sox2 and Oct4 did not result in RAM transcript loss (Figure S9), and RAM transcript loss did not accompany RAM protein loss during neural differentiation (Figure 1F). Characterization of RAM regulation focused on Sox2 rather than Oct4, since inhibition of later results in overt toxicity. The reduction in RAM expression resulting from Sox2 suppression was reversed by addition of a proteasome

(D) RAM siRNA-induced directional transcript changes for ESC- or neural-specific gene sets (see Experimental Procedures for a sets description).

(E) Boxplot representation of (D). p values (false discovery rate [FDR]) are log10 transformed and shown below or above axis for down- or upregulated gene sets, respectively.

(F) Scatterplot of expression changes at transcript and polysome level.

(G) Boxplot of transcript changes of proteins unchanged (unsig.), downregulated, or upregulated in mass spectrometry analysis (Figure S6). Gene set tests performed with ROAST (see Experimental Procedures). $^{**}p < 0.01$, $^{***}p < 0.001$. Boxplot whiskers span from the 5th to 95th percentiles.

See also Figures S5–S7.

OPEN
ACCESS
CellPress

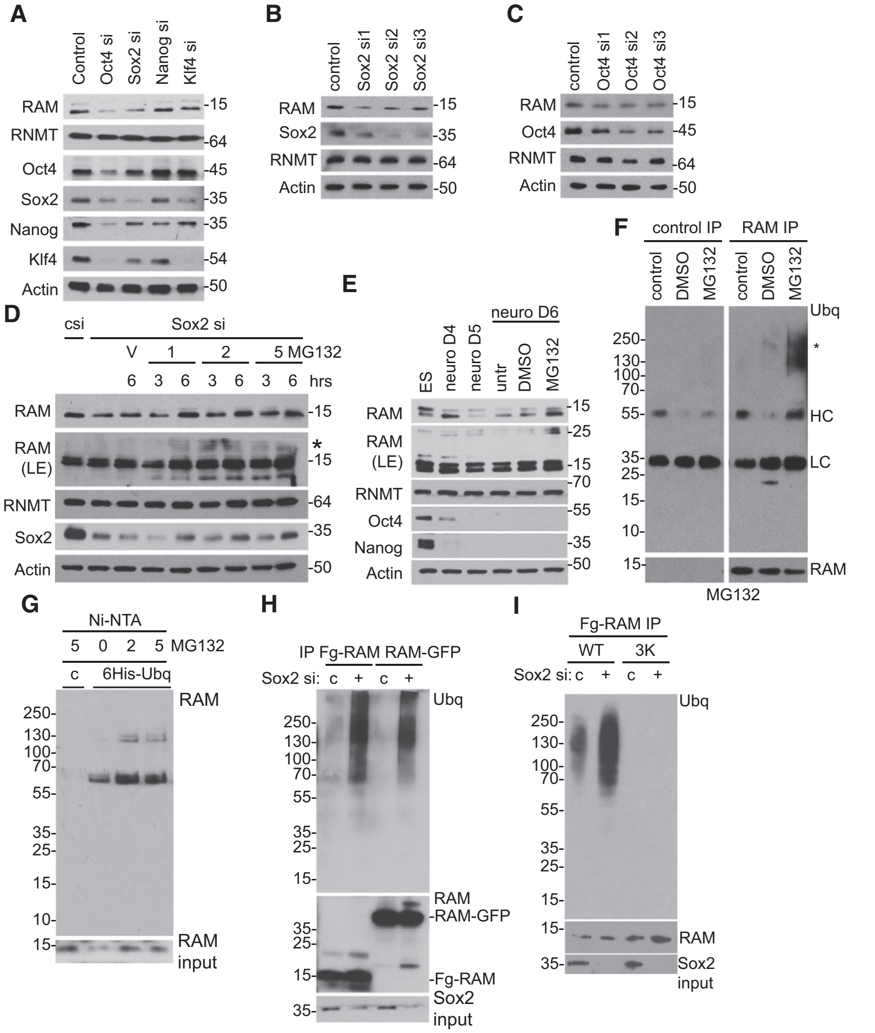

Figure 5. RAM Is Ubiqutinated and Degraded by the Proteasome

(A–C) Expression of Oct4, Sox2, Klf4, and Nanog inhibited by transfection of siRNA pools (A) or independent siRNAs (B and C) for 72 hr. Proteins were detected by WB.

(D) ESCs were transfected with Sox2 siRNA for 72 hr, then 1, 2, or 5 μM MG132 was added for the time indicated. WB analysis is shown. LE indicates long exposure, and the asterisk (*) indicates high-molecular-weight protein.

(E) ESCs subject to neural differentiation. On day 6, cells were treated with 2 μM MG132 or DMSO control for 3 hr.

(F) Neural differentiation on day 7. Immunoprecipitations were performed on cell extracts using anti-RAM or control antibodies. Asterisk (*) indicates high-molecular-weight ubiquitinated proteins detected by WB. LC, light chain; HC, heavy chain.

(G) ESCs expressing 6His-ubiquitin (6His-Ubq) or control (c) incubated in 2 or 5 μM MG132 for 4 hr. Extracts subject to Ni-NTA agarose pull-down and WB.

(H) ESC:Fg-RAM or RAM-GFP transfected with Sox2 siRNA for 72 hr. Fg-RAM and RAM-GFP were purified via tags. RAM and high-molecular-weight ubiquitinated protein was detected by WB.

(I) As in (H), except Fg-RAM WT and 3K were expressed.

See also Figure S9.

inhibitor MG132 (Figure 5D). Treatment with MG132 resulted in high molecular weight smear visible in the RAM western blot, consistent with an unstable pool of modified RAM. Furthermore, loss of RAM during neural differentiation was rescued by treatment with MG132, accompanied by high-molecular-weight RAM protein (Figure 5E).

Since covalent linkage of ubiquitin can mark proteins for degradation, we investigated whether RAM is ubiquitinated prior to degradation. RAM IPs from day 7 neural precursors included high-molecular-weight ubiquitinated protein, which increased following proteasome inhibition, consistent with an unstable pool of RAM-ubiquitin (Figure 5F). RAM was confirmed to be ubiquitinated by purification in denaturing conditions covalently linked to 6His-ubiquitin via nickel-charged resin (Figure 5G). When Sox2 expression was inhibited by siRNA transfection, RAM ubiquitination increased (Figure 5H). This result was observed with Fg-RAM and RAM-GFP, which were transfected and purified via their tags. Since ubiquitin conjugates to lysine residues, point mutations were made to map ubiquitination sites (K10N, K24R, and K31R; RAM 3K). K to R mutations are usually made to inhibit ubiquitination, but the K10N mutation was used, since this is a SNP. RAM 3K exhibited reduced ubiquitination, consistent with ubiquitin being conjugated to one or more of the mutated lysine residues (Figure 5I). Mapping RAM ubiquitination using single K mutants was attempted. However, some of these mutants were toxic and poorly expressed, precluding robust conclusions being drawn.

ERK1/2 Phosphorylation of RAM S36 Promotes Ubiquitination

We characterized the signaling pathways that trigger RAM ubiquitination and degradation during differentiation. We observed that RAM is phosphorylated on serine 36 (S36) and investigated the impact of this modification on ubiquitination and degradation. ESC lines were created expressing Fg-RAM WT and S36A to ablate phosphorylation. Basal levels of Fg-RAM WT and S36A-ubiquitination were detected in untreated controls (Figure 6A). On MG132 treatment, Fg-RAM WT and S36A-ubiquitin increased, indicating that these ubiquitin conjugates are degraded by the proteasome in ESCs. Despite being expressed equivalently, Fg-RAM S36A exhibited reduced ubiquitination compared to WT, indicating that S36 phosphorylation is important (albeit not essential) for this modification. Note that the DMSO used as a vehicle control reduces basal RAM ubiquitination, the reasons for which are unclear, but DMSO is routinely observed to influence enzyme function.

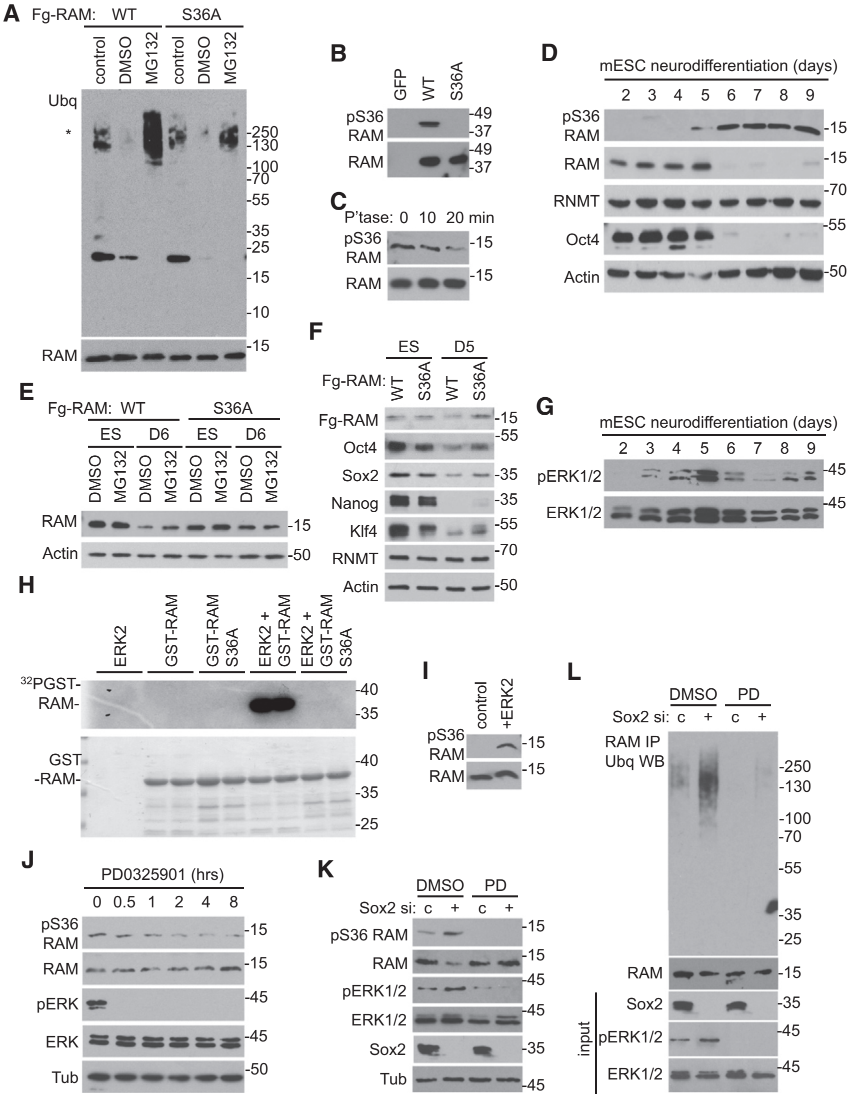

Figure 6. ERK-Dependent S36 Phosphorylation Targets RAM for Degradation during Differentiation

(A) Fg-RAM WT or S36A purified from ESCs treated with 5 μM MG132, DMSO control, or untreated for 3 hr. RAM and ubiquitinated protein detected by WB.

(B) RAM-GFP WT and S36A and GFP alone (purified) and WBs were performed to detect pS36 RAM and total RAM.

(C) ESC extracts treated with lambda phosphatase for the time indicated, and pS36 RAM and total RAM were detected.

(D) ESCs subjected to neural differentiation protocol. Extracts were analyzed by WB on the days indicated.

(E) Fg-RAM WT and S36A expressed in ESCs. ESCs and day 6 neural precursors, pre-treated with 2 μM MG132 or DMSO control for 3 hr, were analyzed by WB.

(F) As in (E), except expression of Oct4, Nanog, and Sox2 was analyzed in day 5 neural precursors.

(G) Expression of pERK1/2 and ERK1/2 analyzed by WB during neural differentiation.

(H) 50 ng ERK2 incubated with 1 μg GST-RAM WT or S36A and [32]PATP for 1 hr at 37°C. Phosphorylated protein was detected by film. GST-RAM was visualized by Coomassie staining.

(I) 10 ng GST-RAM subject to in vitro kinase assay with ERK2 or control analyzed by WB.

(J) Day 5 neurodifferentiated cells treated with 1 μM PD0325901 for the time indicated and proteins detected by WB.

(K) ESCs transfected with Sox2 siRNA or control for 72 hr and treated with 1μM PD0325901or DMSO for 12 hr. Extracts were analyzed by WB.

(L) As in (K), except cells were treated with 2 μM MG132 for 2 hr prior to WB analysis. Top: RAM was immunoprecipitated and ubiquitin detected.

See also Figure S10.

In order to characterize RAM S36 phosphorylation, an antibody was raised against pS36, a RAM peptide phosphorylated on serine 36. The pS36 RAM antibody was phospho-specific, since it detected RAM-GFP wild-type (WT) and not S36A (Figure 6B), and the signal for endogenous RAM diminished following phosphatase treatment (Figure 6C). Since S36 is important for RAM ubiquitination, we investigated if RAM pS36 levels alter during neural differentiation. RAM pS36 was at the limit of detection until day 5, after which it was present, coincident with reduced total RAM levels (Figures 6D and S2A). Since the S36A mutation results in decreased RAM ubiquitination, its effect on protein stabilization during differentiation was investigated. Expression of Fg-RAM S36A was equivalently to that observed in WT ESCs (Figure 6E). However, on day 6 of neural differentiation, Fg-RAM WT expression was reduced and partially rescued by proteasome inhibition, whereas RAM S36A was stable during differentiation (Figure 6E). Consistent with the expression of the markers of pluripotency, Oct4 and Sox2, being dependent on RAM, RAM S36A ameliorated their repression during neural differentiation (Figure 6F), similar to the effect of RAM-GFP (Figure 4).

RAM S36 lies in a potential recognition motif for several kinases, including ERK1/2, on which we focused, since it suppresses pluripotent gene expression (Hamilton and Brickman, 2014; Ying et al., 2003). Consistent with previous observations, active phospho-ERK1/2 peaked at day 5 during neural differentiation, when RAM pS36 and RAM degradation was first observed (Figure 6G) (Pickford et al., 2011). Recombinant ERK 2 phosphorylated recombinant RAM WT, but not RAM S36A, directly in vitro (Figure 6H). The pS36 antibody was further validated by the specific detection of ERK2-phosphorylated RAM (Figure 6I). In ESCs, inhibition of ERK activation using 1 μM PD0325901 on day 5 of neural differentiation inhibited RAM phosphorylation (Figure 6J). Sox2 inhibition resulted in increased RAM S36 phosphorylation, decreased expression (Figure 6K), and increased ubiquitination (Figure 6L), and PD0325901 reversed these effects.

Other kinases predicted based on substrate motif to be potential RAM S36 kinases (JNK, CDK1-cyclin B, CDK2-cyclin A1, and

CDK3-cyclin E) did not detectably phosphorylate RAM in vitro and are therefore unlikely to phosphorylate RAM in cells (Figure S10). However, since ERK1/2 activity is not maintained at high levels during differentiation, other kinases may phosphorylate RAM S36 at this time.

RNMT-RAM Upregulation Has a Role in Reprogramming

Since RAM is important for the expression of pluripotency-associated genes in ESCs, we investigated whether induction of pluripotency or ‘‘reprogramming’’ utilizes RAM. Initially, RNMT and RAM expression in ESCs, MEFs (mouse embryonic fibroblasts), and iPSCs (induced pluripotent stem cells) was compared. Strain 129/Ola MEFs were transduced with vectors expressing Oct4, Klf4, Sox2, and c-Myc (Woltjen et al., 2009). Reprogrammed colonies (iPSCs) became visible as tightly packed clusters of embryonic stem-like cells, staining alkaline phosphatase (AP) positive and expressing Oct4, Sox2, c-Myc, and Nanog (Figures 7A, 7B, and 7D). In comparison to ESCs, MEFs contained less RNMT and RAM, and reprogramming to iPSCs restored expression of both (Figure 7B). The transcript level for RNMT and RAM was lower in MEFs than in ESCs and iPSCs, suggesting that a component of RNMT and RAM suppression is at the transcript level (Figure 7D). The same trend was observed with strain C57/BL6 MEFs and iPSCs (data not shown). RNMT and RAM expression was also reduced in human fibroblasts (hFs) compared to human ESCs (hESCs) and restored in human iPSCs (Figure 7C). As with MEFs, RNMT and RAM transcript level was reduced in human fibroblasts (Figure 7E).

We investigated the function of RNMT and RAM in MEF reprogramming. Reprogramming was induced by expression of Oct4, Sox2, Klf4, and c-Myc, which resulted in increased RAM expression after 5 days (Figure 7F). During reprogramming, RAM or RNMT expression was increased by overexpression of RAM-GFP or HA-RNMT and RAM-GFP on day 3 or 4, resulting in increased reprogramming efficiency (Figure 7G). Conversely, inhibition of RAM or RNMT expression by transfection of siRNA resulted in a reduction in iPSC colony formation (Figure 7H).

DISCUSSION

Differentiation of ESCs employs extensive coordinated regulation of transcription, RNA processing, translation, and protein modification to achieve the functional proteomes required of each lineage (Dunn et al., 2014; Lu et al., 2009; Sampath et al., 2008; Tanabe et al., 2014). The cap is a potent mRNA modification with the potential to coordinate the expression of large cohorts of genes. We report that regulation of the mRNA cap methyltransferase RNMT-RAM makes a critical contribution to the gene expression changes required of differentiation and reprogramming. In vertebrates, the cap methyltransferase consists of RNMT, the enzymic subunit, and RAM, the activator subunit. High levels of RAM are found in ESCs, whereas in most murine organs, there is reduced or minimal expression. Focusing on the neural system, high levels of RNMT are present in brain tissue, cortical neurons, and in-vitro-differentiated neural progenitors, whereas there are minimal levels of RAM.

RAM Function in ESCs

We addressed the biological significance of high RAM expression and cap methyltransferase activity in ESCs. In ESCs, $\sim$20% of genes analyzed were dependent on RAM for expression at the transcript level, including core pluripotency transcription factors, and pluripotency-associated transcriptional regulators and chromatin remodelers. The mRNA cap protects transcripts from degradation during transcription, and the cap methyltransferases have been demonstrated to promote transcription (Topisirovic et al., 2011). In addition, some transcript-level control may be a result of the indirect effects of RAM on transcriptional regulators.

In contrast to its specific effect on transcripts, in ESCs, RAM is a non-specific, global activator of translation. In response to RAM suppression, there was a significant loss of polysomal transcripts, but sequencing analysis revealed remarkably little selectivity to these genes. The mRNA cap binds to eIF4F, which recruits the transcript to the ribosome. However, our proteomic analysis revealed that RAM also regulates expression of translation factors, ribosomal subunits, and tRNA synthetases and thus may also regulate global translation indirectly.

Oct4 and Sox2 are two transcription factors required for pluripotency, and their repression is required for many programs of differentiation. In this paper, we demonstrate that Oct4 and Sox2 are RAM responsive. At the protein level, Oct4 and Sox2 are repressed in ESCs 2 days following transfection with RAM siRNA, and they are maintained during neural differentiation if RAM-GFP is expressed to prevent RAM repression. What is the mechanism of Oct4 and Sox2 regulation by RAM? We demonstrate that expression of Oct4 transcripts is dependent on RAM. In addition, Oct4 protein has a relatively short half-life, and therefore, although RAM inhibits polysome loading of all transcripts equivalently, proteins with short half-life are likely to be particularly responsive to translational inhibition (Buckley et al., 2012). Oct4 and Sox2 are also likely to be indirectly inhibited by suppression of RAM during differentiation. Repression of RAM inhibits expression of a large cohort of pluripotency-associated factors that may promote differentiation, repressing Oct4 and Sox2 as part of the process.

Gene Specificity of RAM

RAM not only activates RNMT catalytic activity but also recruits RNA to the methyltransferase via its RNA-binding domain. Prior to this study, RAM was thought to be a constitutive factor present on every RNMT protein, and therefore, whether the RNA binding domain has sequence specificity was not at the forefront of investigation (Gonatopoulos-Pournatzis et al., 2011). Now that we recognize that there is a loss of RNMT-RAM heterodimers and gain of RNMT without RAM (probably monomers) during differentiation, the nature of RAM-RNA binding specificity becomes key to understanding its function. Since sequencing analysis indicated that a subset of transcripts were dependent on RAM for expression, this suggests that RAM does have sequence, motif, or chromatin context specificity.

RAM Expression Control

The kinase ERK1/2 is upregulated during differentiation and is an established suppressor of pluripotency-associated genes

OPEN ACCESS
CellPress

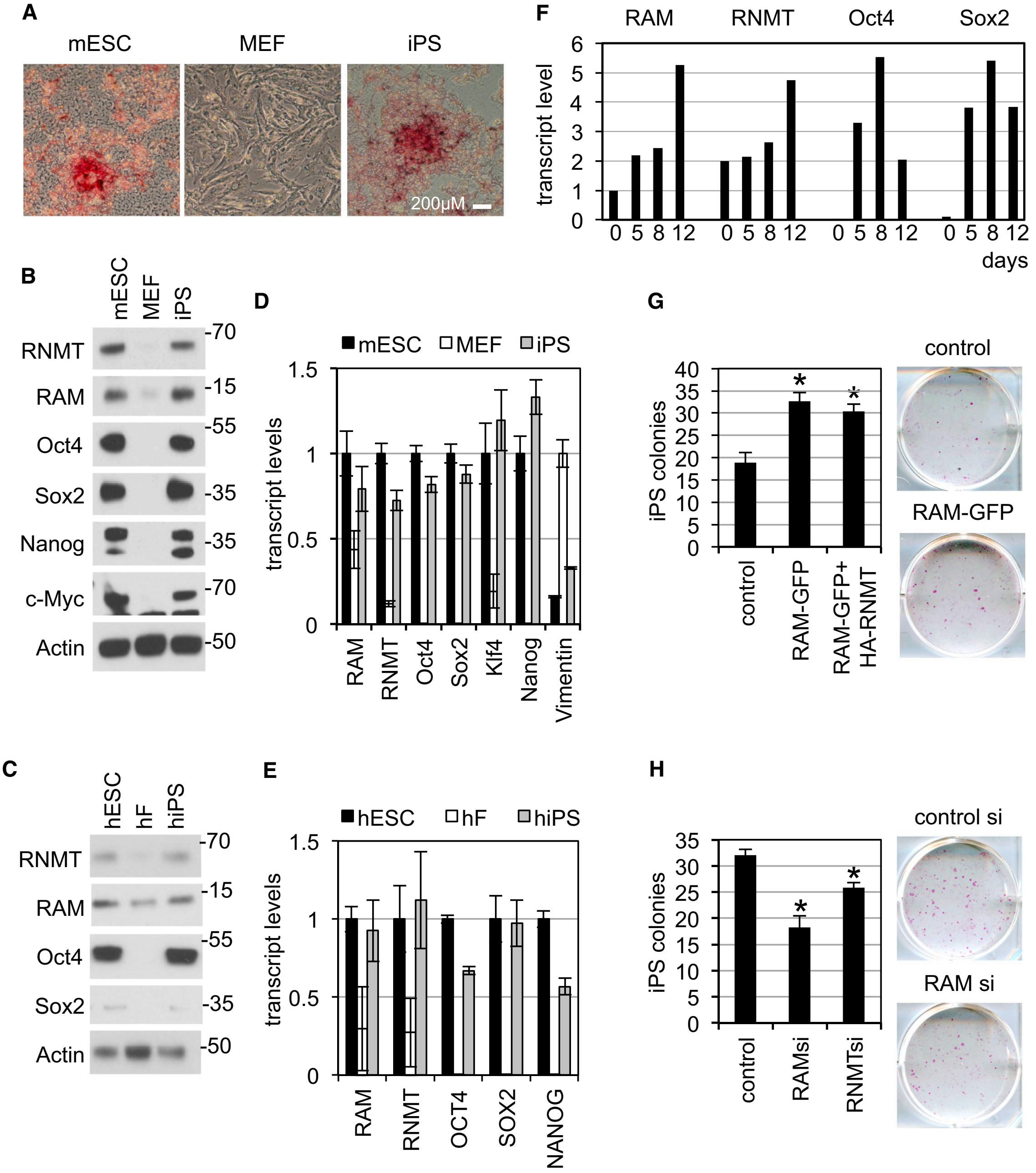

Figure 7. High Levels of RNMT-RAM Are Required for Pluripotency

(A) Murine ESC, MEFs, and iPSCs were AP stained. Scale bar, 200 μm.

(B) Proteins analyzed by WB.

(C) Proteins analyzed by WB in extracts from human ESCs (hESC), human fibroblasts (hF), and human iPSCs (hiPS).

(D and E) Average transcript level for three independent experiments detected by RT-PCR in murine ESCs, MEFs, and iPSCs (D) and hESCs, hFs, and iPSCs (E).

(F) Oct4, Klf4, Sox2, and c-Myc expressed in MEFs and transcript levels detected on the days indicated.

(G and H) After 3–4 days, cells were also transfected with vectors to express RAM-GFP, or RAM-GFP and HA-RNMT (G) or cells were transfected with RNMT or RAM siRNA (H). After 14 days, the relative number of colonies with ESC-like morphology and AP-positive colonies was scored. Mean and SD for three independent experiments are shown. t test p values calculated relative to control (*$p \leq 0.05$). Representative wells of AP-stained iPSC colonies.

(Burdon et al., 1999; Hamilton and Brickman, 2014; Stavridis et al., 2007). ERK1/2 phosphorylates RAM S36, triggering ubiquitination and proteosomal degradation. Phospho-S36 may recruit an E2/E3 ligase that ubiquitinates RAM or inhibits interaction with a deubiquitinating enzyme. Our findings are consistent with ERK1/2-dependent repression of RAM being

an important contributor to the mechanism by which it represses pluripotency-associated genes (Burdon et al., 1999).

ERK1/2 specifically targets RAM for degradation, leaving RNMT intact. RNMT and RAM interact with high affinity, and RAM monomers are not readily observed in cells, and therefore, the specific targeting of RAM is mechanistically surprising. However, our in vitro kinase assays revealed that ERK2 only phosphorylates monomeric RAM and not RAM in complex with RNMT. In the RNMT-RAM structure, RAM S36 is partially buried in RNMT and therefore inaccessible as an ERK1/2 substrate (unpublished data). Thus, ERK1/2 is able to specifically target newly synthesized monomeric RAM for degradation, leaving existing RNMT-RAM and nascent RNMT monomers intact.

RAM-ESC Gene Regulatory Network Feedback Provides Robustness to Pluripotency

Here, we describe a positive feedback loop between RNMT-RAM and pluripotency-associated gene expression, which we postulate provides robustness to pluripotency and differentiation. High levels of RAM result in the expression of a large cohort of pluripotency associated genes. Should pluripotency-associated transcript levels fluctuate in ESCs, high levels of RNMT-RAM will maintain their expression. However, when the trigger for differentiation is sustained, these transcripts may fall below a threshold at which RNMT-RAM cannot maintain their expression. At this point, ERK1/2 activity increases, RAM becomes phosphorylated, ubiquitinated, and degraded, and RAM-dependent pluripotency-associated genes are repressed, permitting differentiation. Thus, RNMT-RAM is a critical component of the pluripotency network.

EXPERIMENTAL PROCEDURES

ESC Culture

46C ESCs (expressing Sox1-GFP) derived from strain 129Ola mice were cultured on 0.1% gelatin-coated dishes in Glasgow minimal essential medium (Sigma), 10% knockout serum replacement, 1% modified Eagle's medium (MEM) non-essential amino acids, 1 mM sodium pyruvate (Life Technologies), 0.1 mM 2-mercaptoethanol (Sigma), and 100 U/ml recombinant human leukemia inhibitory factor). hiPS4 and SA181 human ESC lines purchased from Cellartis and maintained in DEF-CS (Cellartis). Human neural differentiation was performed as described previously (Chambers et al., 2009), except that cells were seeded at a density of 6 × 10^4 cells/cm^2 on Matrigel (20 μg/cm^2) and grown for 48 hrs in cell medium before switching to differentiation media. Murine neural differentiation was performed as described previously (Stavridis et al., 2007; Ying et al., 2003). 0.5–1.5 × 10^4/cm^2 ESCs were plated on 0.1% gelatin-coated dishes in N2B27 (DMEM/F12; Gibco) supplemented with modified N2 (25 μg/ml insulin, 100 μg/ml apo-transferrin, 6 ng/ml progesterone, 16 μg/ml putrescine, 30 nM sodium selenite and 50 μg/ml BSA fraction V; Gibco). Medium was renewed every 2 days.

Primary Neural Cell Culture

Primary cortical neurons established from E16 mice (strain C57Bl) are a mixed population of terminally differentiated neurons from the cortex. Primary neuronal cells were plated onto 10 μg/ml poly-L-lysine-coated flasks and maintained in Neurobasal medium with B27 supplement (Life Technologies), 2 mM glutamine, 100 U/ml penicillin, and 100 μg/ml streptomycin (Lonza). Astrocytes are dividing cells of the astroglial line, established from P2. Astrocytes were maintained in DMEM/10% fetal calf serum until confluent.

Transfections

1 × 10^5 cells transfected with 200 pmol siRNA or non-targeting controls (siGenome, Dharmacon) using Lipofectamine 2000 (Invitrogen). 1 × 10^6 ESCs in a 10-cm dish transfected with 4 μg pPyPCAGIP plasmids using Fugene HD (Promega) or Lipofectamine 2000 (Life Technologies). 1 μg/ml puromycin was used for selection.

Reprogramming

1.5 × 10^5 MEFs were plated on a six-well plate well. 1.5 μg PB-TAP IRI 2OKSMimO (expressing Oct4, Klf4, Sox2, c-Myc), AG-rtTA (reverse tetracycline transactivator), and HyPBase (transposase) was transfected using Fugene HD (Invitrogen) (Kaji et al., 2009; Woltjen et al., 2009). At day 3 or 4, cells were transfected with 200 pmol RAM siRNA, 1 μg pPyPCAGIP RAM-GFP, or 0.5 μg pPyPCAGIP RAM-GFP and 0.5 μg pPyPCAGIP HA-RNMT. Induced pluripotent cell colony number was counted following AP staining after 14 days of reprogramming.

Statistics

Statistical methodologies are reported in figure legends and in proteomics and RNA-seq experimental procedures.

See Supplemental Experimental Procedures for polysome profile and RNA-seq analysis, proteomics analysis, molecular biology, immunological techniques, kinase assay, and description of expression construct.

ACCESSION NUMBERS

The accession number for the sequencing data reported in this paper is GEO: GSE81849.

SUPPLEMENTAL INFORMATION

Supplemental Information includes Supplemental Experimental Procedures, ten figures, and five tables and can be found with this article online at http://dx.doi.org/10.1016/j.celrep.2016.06.089.

AUTHOR CONTRIBUTIONS

L.G., O.S., M.P.S., and V.H.C. designed and performed experiments and wrote the manuscript. L.D., T.G.-P., R.W., L.W., S.W., and M.P. performed experiments.

ACKNOWLEDGMENTS

We thank the Findlay, M.P.S., and V.H.C. lab members. Research was funded by an MRC Senior Fellowship (MR/K024213/1 to V.H.C.), a Lister Institute Prize Fellowship (to V.H.C.), Wellcome Trust PhD studentships (097462/Z/11/Z to L.G. and O.S.), and the University of Dundee Human Pluripotent Stem Cell Facility. Research was supported by Wellcome Institutional Support Fund 097818/Z/11/B, GRE Centre Award 097945/Z/11/Z, and the Division of Signal Transduction Therapy Unit (AstraZeneca, Boehringer-Ingelheim, GlaxoSmithKline, Merck KGaA, Janssen Pharmaceutica, and Pfizer).

Received: October 10, 2015
Revised: May 24, 2016
Accepted: June 24, 2016
Published: July 21, 2016

REFERENCES

Aregger, M., and Cowling, V.H. (2012). E2F1-dependent methyl cap formation requires RNA pol II phosphorylation. Cell Cycle *11*, 2146–2148.

Aregger, M., and Cowling, V.H. (2013). Human cap methyltransferase (RNMT) N-terminal non-catalytic domain mediates recruitment to transcription initiation sites. Biochem. J. *455*, 67–73.

Aregger, M., Kaskar, A., Varshney, D., Fernandez-Sanchez, M.E., Inesta-Vaquera, F.A., Weidlich, S., and Cowling, V.H. (2016). CDK1-cyclin B1 activates RNMT, coordinating mRNA cap methylation with G1 phase transcription. Mol. Cell *61*, 734–746.

Aubert, J., Stavridis, M.P., Tweedie, S., O'Reilly, M., Vierlinger, K., Li, M., Ghazal, P., Pratt, T., Mason, J.O., Roy, D., and Smith, A. (2003). Screening for mammalian neural genes via fluorescence-activated cell sorter purification of neural precursors from Sox1-gfp knock-in mice. Proc. Natl. Acad. Sci. USA *100* (*Suppl 1*), 11836–11841.

Bickmore, W.A., and Zaret, K.S. (2010). Altered states: how gene expression is changed during differentiation. Curr. Opin. Genet. Dev. *20*, 467–469.

Boroviak, T., Loos, R., Lombard, P., Okahara, J., Behr, R., Sasaki, E., Nichols, J., Smith, A., and Bertone, P. (2015). Lineage-Specific Profiling Delineates the Emergence and Progression of Naive Pluripotency in Mammalian Embryogenesis. Dev. Cell *35*, 366–382.

Buckley, S.M., Aranda-Orgilles, B., Strikoudis, A., Apostolou, E., Loizou, E., Moran-Crusio, K., Farnsworth, C.L., Koller, A.A., Dasgupta, R., Silva, J.C., et al. (2012). Regulation of pluripotency and cellular reprogramming by the ubiquitin-proteasome system. Cell Stem Cell *11*, 783–798.

Burdon, T., Stracey, C., Chambers, I., Nichols, J., and Smith, A. (1999). Suppression of SHP-2 and ERK signalling promotes self-renewal of mouse embryonic stem cells. Dev. Biol. *210*, 30–43.

Chambers, S.M., Fasano, C.A., Papapetrou, E.P., Tomishima, M., Sadelain, M., and Studer, L. (2009). Highly efficient neural conversion of human ES and iPS cells by dual inhibition of SMAD signaling. Nat. Biotechnol. *27*, 275–280.

Chen, X., Xu, H., Yuan, P., Fang, F., Huss, M., Vega, V.B., Wong, E., Orlov, Y.L., Zhang, W., Jiang, J., et al. (2008). Integration of external signaling pathways with the core transcriptional network in embryonic stem cells. Cell *133*, 1106–1117.

Chu, C., and Shatkin, A.J. (2008). Apoptosis and autophagy induction in mammalian cells by small interfering RNA knockdown of mRNA capping enzymes. Mol. Cell. Biol. *28*, 5829–5836.

Cole, M.D., and Cowling, V.H. (2009). Specific regulation of mRNA cap methylation by the c-Myc and E2F1 transcription factors. Oncogene *28*, 1169–1175.

Cowling, V.H., and Cole, M.D. (2007). The Myc transactivation domain promotes global phosphorylation of the RNA polymerase II carboxy-terminal domain independently of direct DNA binding. Mol. Cell. Biol. *27*, 2059–2073.

Cox, J., and Mann, M. (2008). MaxQuant enables high peptide identification rates, individualized p.p.b.-range mass accuracies and proteome-wide protein quantification. Nat. Biotechnol. *26*, 1367–1372.

Dunn, S.J., Martello, G., Yordanov, B., Emmott, S., and Smith, A.G. (2014). Defining an essential transcription factor program for naïve pluripotency. Science *344*, 1156–1160.

Fernandez-Sanchez, M.E., Gonatopoulos-Pournatzis, T., Preston, G., Lawlor, M.A., and Cowling, V.H. (2009). S-adenosyl homocysteine hydrolase is required for Myc-induced mRNA cap methylation, protein synthesis, and cell proliferation. Mol. Cell. Biol. *29*, 6182–6191.

Glover-Cutter, K., Kim, S., Espinosa, J., and Bentley, D.L. (2008). RNA polymerase II pauses and associates with pre-mRNA processing factors at both ends of genes. Nat. Struct. Mol. Biol. *15*, 71–78.

Gonatopoulos-Pournatzis, T., and Cowling, V.H. (2014a). Cap-binding complex (CBC). Biochem. J. *457*, 231–242.

Gonatopoulos-Pournatzis, T., and Cowling, V.H. (2014b). RAM function is dependent on Kapβ2-mediated nuclear entry. Biochem. J. *457*, 473–484.

Gonatopoulos-Pournatzis, T., Dunn, S., Bounds, R., and Cowling, V.H. (2011). RAM/Fam103a1 is required for mRNA cap methylation. Mol. Cell *44*, 585–596.

Hamilton, W.B., and Brickman, J.M. (2014). Erk signaling suppresses embryonic stem cell self-renewal to specify endoderm. Cell Rep. *9*, 2056–2070.

Jia, J., Zheng, X., Hu, G., Cui, K., Zhang, J., Zhang, A., Jiang, H., Lu, B., Yates, J., 3rd, Liu, C., et al. (2012). Regulation of pluripotency and self- renewal of ESCs through epigenetic-threshold modulation and mRNA pruning. Cell *151*, 576–589.

Jiao, X., Xiang, S., Oh, C., Martin, C.E., Tong, L., and Kiledjian, M. (2010). Identification of a quality-control mechanism for mRNA 5′-end capping. Nature *467*, 608–611.

Jiao, X., Chang, J.H., Kilic, T., Tong, L., and Kiledjian, M. (2013). A mammalian pre-mRNA 5′ end capping quality control mechanism and an unexpected link of capping to pre-mRNA processing. Mol. Cell *50*, 104–115.

Kaji, K., Norrby, K., Paca, A., Mileikovsky, M., Mohseni, P., and Woltjen, K. (2009). Virus-free induction of pluripotency and subsequent excision of reprogramming factors. Nature *458*, 771–775.

Kim, J., Chu, J., Shen, X., Wang, J., and Orkin, S.H. (2008). An extended transcriptional network for pluripotency of embryonic stem cells. Cell *132*, 1049–1061.

Lu, R., Markowetz, F., Unwin, R.D., Leek, J.T., Airoldi, E.M., MacArthur, B.D., Lachmann, A., Rozov, R., Ma'ayan, A., Boyer, L.A., et al. (2009). Systems-level dynamic analyses of fate change in murine embryonic stem cells. Nature *462*, 358–362.

Martí, M., Mulero, L., Pardo, C., Morera, C., Carrió, M., Laricchia-Robbio, L., Esteban, C.R., and Izpisua Belmonte, J.C. (2013). Characterization of pluripotent stem cells. Nat. Protoc. *8*, 223–253.

Mukherjee, C., Patil, D.P., Kennedy, B.A., Bakthavachalu, B., Bundschuh, R., and Schoenberg, D.R. (2012). Identification of cytoplasmic capping targets reveals a role for cap homeostasis in translation and mRNA stability. Cell Rep. *2*, 674–684.

Pickford, C.E., Holley, R.J., Rushton, G., Stavridis, M.P., Ward, C.M., and Merry, C.L. (2011). Specific glycosaminoglycans modulate neural specification of mouse embryonic stem cells. Stem Cells *29*, 629–640.

Pijnappel, W.W., Esch, D., Baltissen, M.P., Wu, G., Mischerikow, N., Bergsma, A.J., van der Wal, E., Han, D.W., Bruch, Hv., Moritz, S., et al. (2013). A central role for TFIID in the pluripotent transcription circuitry. Nature *495*, 516–519.

Ponnusamy, M.P., Deb, S., Dey, P., Chakraborty, S., Rachagani, S., Senapati, S., and Batra, S.K. (2009). RNA polymerase II associated factor 1/PD2 maintains self-renewal by its interaction with Oct3/4 in mouse embryonic stem cells. Stem Cells *27*, 3001–3011.

Sampath, P., Pritchard, D.K., Pabon, L., Reinecke, H., Schwartz, S.M., Morris, D.R., and Murry, C.E. (2008). A hierarchical network controls protein translation during murine embryonic stem cell self-renewal and differentiation. Cell Stem Cell *2*, 448–460.

Shuman, S. (2015). RNA capping: progress and prospects. RNA *21*, 735–737.

Stavridis, M.P., Lunn, J.S., Collins, B.J., and Storey, K.G. (2007). A discrete period of FGF-induced Erk1/2 signalling is required for vertebrate neural specification. Development *134*, 2889–2894.

Stunnenberg, H.G., Vermeulen, M., and Atlasi, Y. (2015). Developmental biology. A Me6Age for pluripotency. Science *347*, 614–615.

Tahmasebi, S., Alain, T., Rajasekhar, V.K., Zhang, J.P., Prager-Khoutorsky, M., Khoutorsky, A., Dogan, Y., Gkogkas, C.G., Petroulakis, E., Sylvestre, A., et al. (2014). Multifaceted regulation of somatic cell reprogramming by mRNA translational control. Cell Stem Cell *14*, 606–616.

Takahashi, K., and Yamanaka, S. (2006). Induction of pluripotent stem cells from mouse embryonic and adult fibroblast cultures by defined factors. Cell *126*, 663–676.

Takahashi, K., Tanabe, K., Ohnuki, M., Narita, M., Ichisaka, T., Tomoda, K., and Yamanaka, S. (2007). Induction of pluripotent stem cells from adult human fibroblasts by defined factors. Cell *131*, 861–872.

Tanabe, K., Takahashi, K., and Yamanaka, S. (2014). Induction of pluripotency by defined factors. Proc. Jpn. Acad., Ser. B, Phys. Biol. Sci. *90*, 83–96.

Topisirovic, I., Svitkin, Y.V., Sonenberg, N., and Shatkin, A.J. (2011). Cap and cap-binding proteins in the control of gene expression. Wiley Interdiscip. Rev. RNA *2*, 277–298.

Wang, L., Miao, Y.L., Zheng, X., Lackford, B., Zhou, B., Han, L., Yao, C., Ward, J.M., Burkholder, A., Lipchina, I., et al. (2013). The THO complex regulates pluripotency gene mRNA export and controls embryonic stem cell self-renewal and somatic cell reprogramming. Cell Stem Cell *13*, 676–690.

Wang, L., Du, Y., Ward, J.M., Shimbo, T., Lackford, B., Zheng, X., Miao, Y.L., Zhou, B., Han, L., Fargo, D.C., et al. (2014). INO80 facilitates pluripotency gene activation in embryonic stem cell self-renewal, reprogramming, and blastocyst development. Cell Stem Cell *14*, 575–591.

Woltjen, K., Michael, I.P., Mohseni, P., Desai, R., Mileikovsky, M., Hämäläinen, R., Cowling, R., Wang, W., Liu, P., Gertsenstein, M., et al. (2009). piggyBac

transposition reprograms fibroblasts to induced pluripotent stem cells. Nature *458*, 766–770.

Wong, Q.W., Vaz, C., Lee, Q.Y., Zhao, T.Y., Luo, R., Archer, S.K., Preiss, T., Tanavde, V., and Vardy, L.A. (2016). Embryonic stem cells exhibit mRNA isoform specific translational regulation. PLoS ONE *11*, e0143235.

Wongpaiboonwattana, W., and Stavridis, M.P. (2015). Neural differentiation of mouse embryonic stem cells in serum-free monolayer culture. J. Vis. Exp. *99*, e52823.

Ying, Q.L., Stavridis, M., Griffiths, D., Li, M., and Smith, A. (2003). Conversion of embryonic stem cells into neuroectodermal precursors in adherent monoculture. Nat. Biotechnol. *21*, 183–186.

Yu, J., Vodyanik, M.A., Smuga-Otto, K., Antosiewicz-Bourget, J., Frane, J.L., Tian, S., Nie, J., Jonsdottir, G.A., Ruotti, V., Stewart, R., et al. (2007). Induced pluripotent stem cell lines derived from human somatic cells. Science *318*, 1917–1920.

Cell Reports, Volume *16*

Supplemental Information

mRNA Cap Methylation in Pluripotency and Differentiation

Laura Grasso, Olga Suska, Lindsay Davidson, Thomas Gonatopoulos-Pournatzis, Ritchie Williamson, Lize Wasmus, Simone Wiedlich, Mark Peggie, Marios P. Stavridis, and Victoria H. Cowling

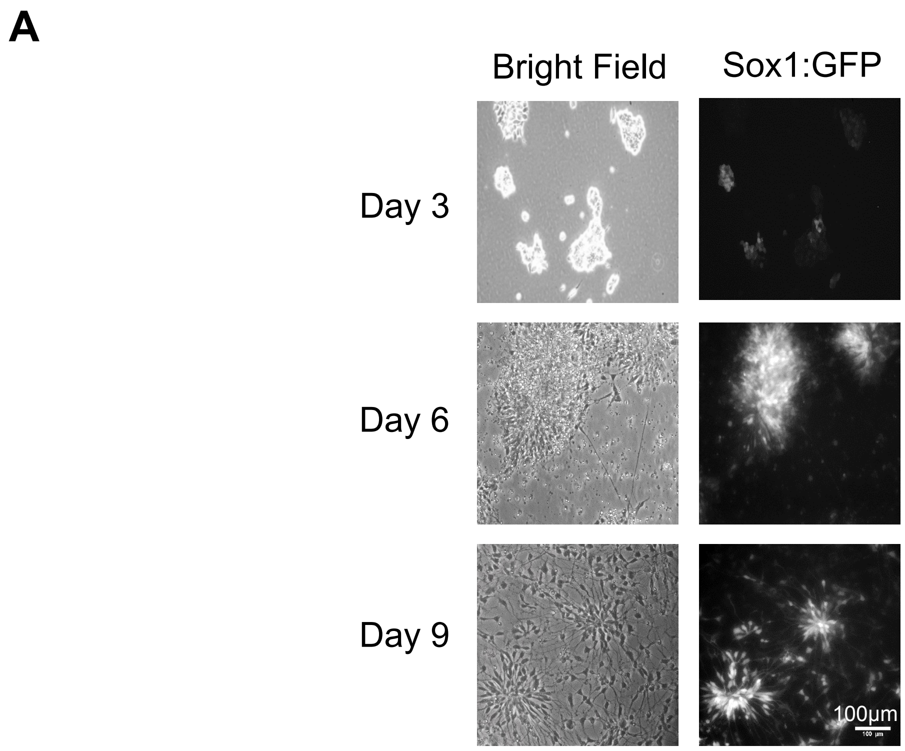

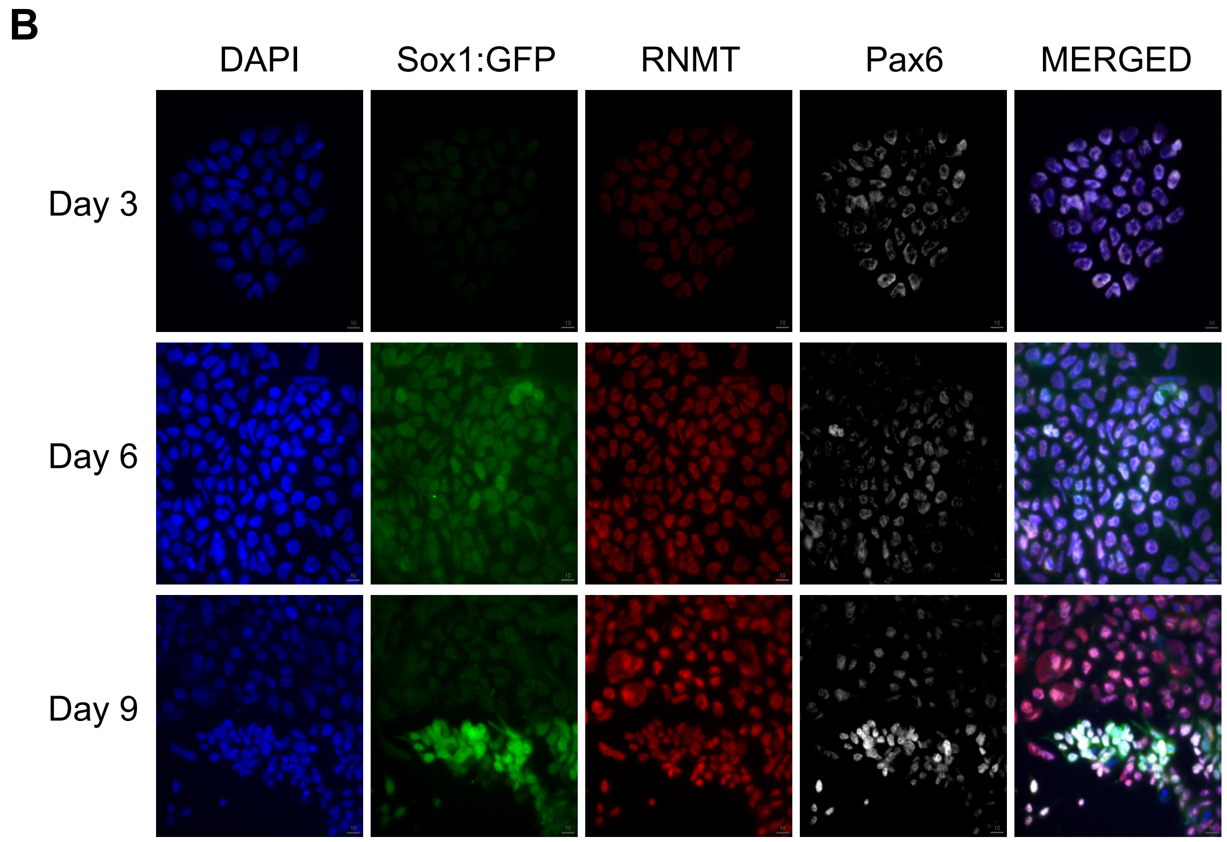

Figure S1 (related to Figure 1) Establishing neural differentiation of ESC
Detection of morphological changes in ES cells during neural differentiation**.** ESCs were seeded in N2B27 media on dishes coated with 0.1% gelatin. Media was changed every second day. Phase-contrast and flourescent images were captured on days 3, 6 and 9. Scale bar measures 100 µm.
b) Immunofluorescence microscopy was used to detect Sox1-GFP and PAX6, markers of neural differentiation. ESCs were seeded in N2B27 media onto coverslip coated with Laminin. Cells were fixed with 4% PFA and stained with the indicated antibodies on days 3, 6 and 9. DAPI staining was used to detect nuclei. High-resolution images were collected using a DeltaVision Restoration; Applied Precision system, using a 40X/1.514 oil Olympus objective lens. Images were processed using OMERO software.

Figure S2

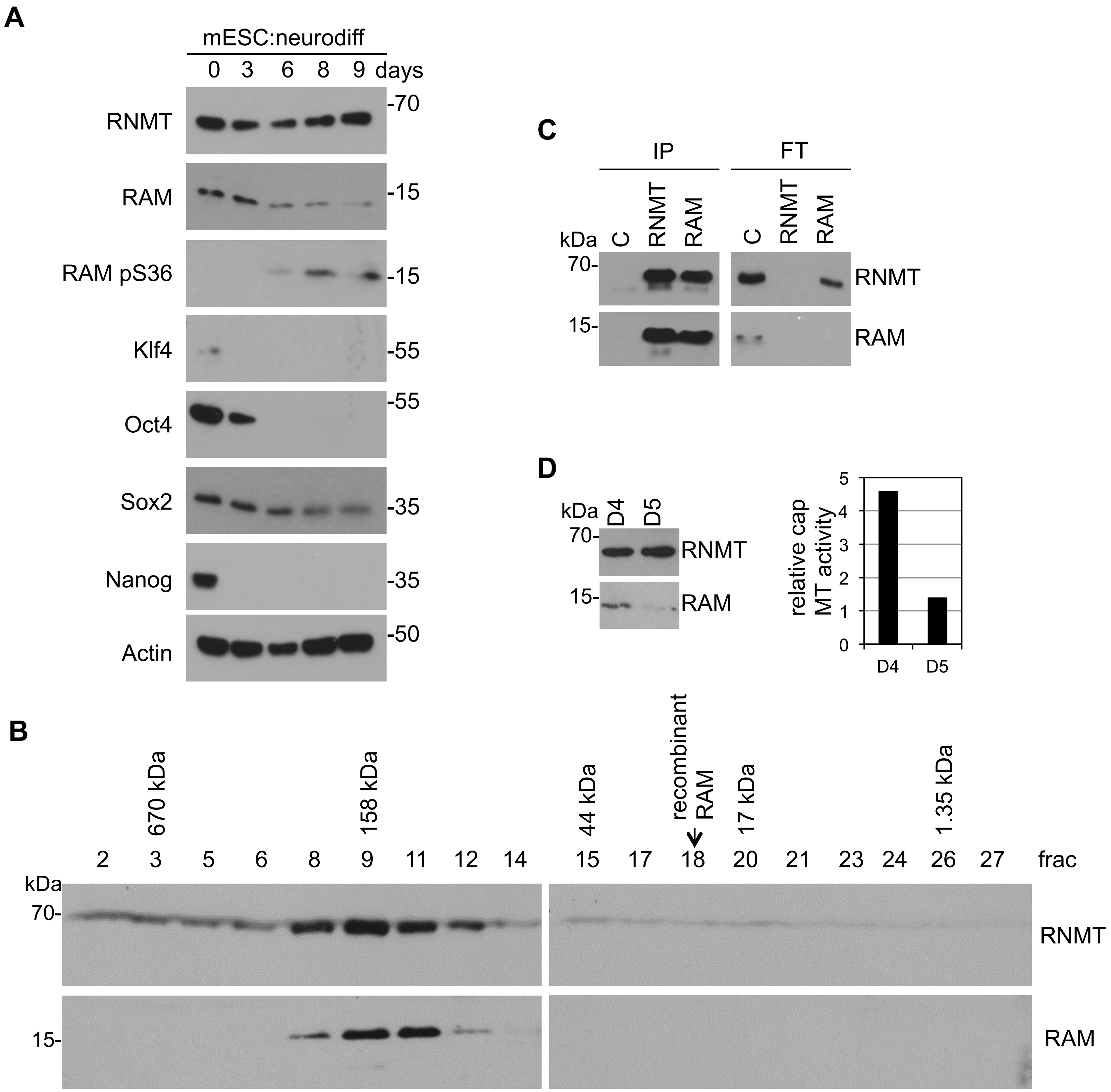

Figure S2 (related to Figure 1) RNMT-RAM in ESCs

a) ESCs were cultured according to a 9-day neural differentiation protocol. Proteins detected by WB. b) 0.5mg ESC extract was separated on a Superdex S200 column in 150 mM NaCl, 50 mM HEPES 0.03 % Brij-35 and 1mM DTT, pH 7.5 and 0.5ml fractions collected. Western blots were performed to detect RNMT and RAM in selected fractions. The migration of markers and recombinant RAM is indicated above blots. c) 10µg ESC extract was subject to immunoprecipitation using anti-GST (c), anti-RNMT and anti-RAM antibodies. Following immuoprecipitation, immunoprecipitates (IP) and residual cell extract (FT) was analysed by western blot for expression of RNMT and RAM. d) The cap methyltransferase activity of ESCs and differentiated cells was analysed by *in vitro* assay. 2µg nuclear extract was incubated with an excess of GpppG-RNA substrate and 100nM S-adenosyl methionine for 7 mins at 30ºC. Products were digested with P1 nuclease and analysed by thin layer chromatography to quantitate m7GpppG-RNA produced. Assays were performed according to (Cowling, 2010).

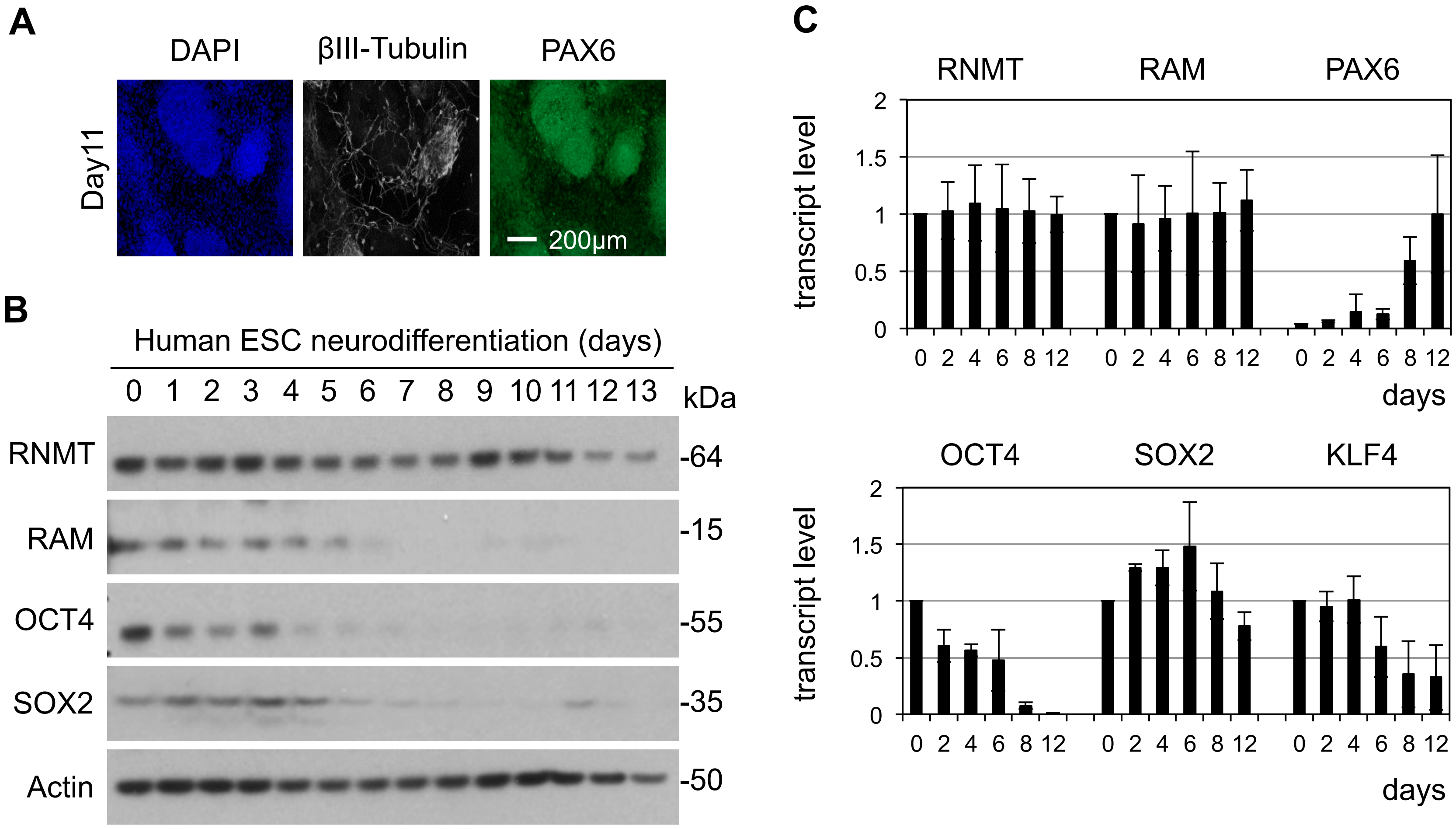

Figure S3 (related to Figure 1) Expression of RAM is suppressed during human ESC neural differentiation
hES cells SA181were subject to neural differentiation protocol. a) Day 11 cultures were analysed for βIII-Tubulin and PAX6 expression by immunofluorescence. b) Cell extracts were analysed by WB. c) Transcript levels were determined by RTPCR relative to Actin expression. Chart represents average and standard deviation of data from three independent experiments.

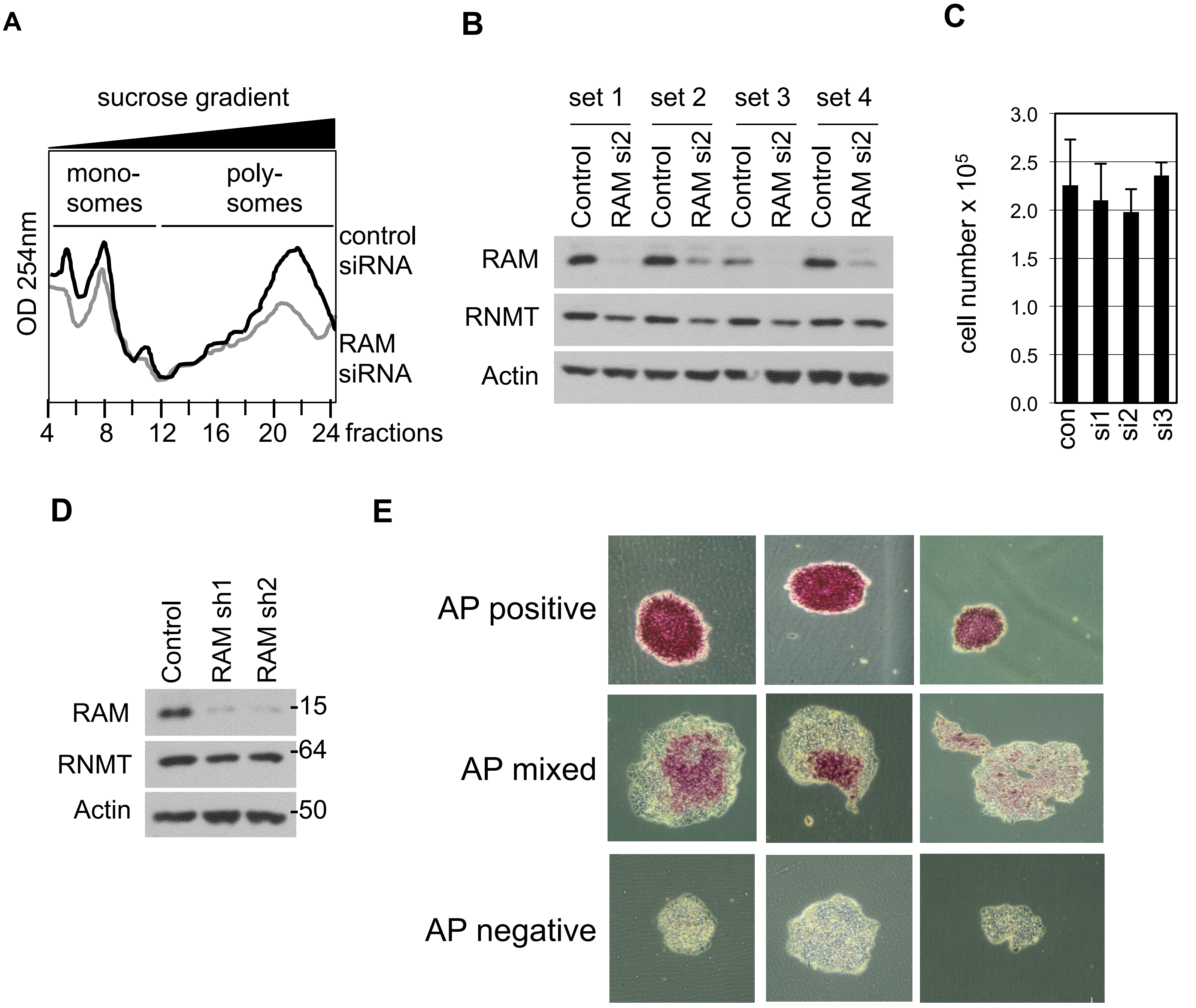

Figure S4 (related to Figure 2) Transfection of ESCs with RAM siRNA and shRNA
ESCs were transfected with a RAM siRNA2 or a non-targetting control. a) After 48hrs RNA-protein complexes were resolved through a sucrose gradient. Protein-RNA complexes detected by absorbance at 254nm. b) ESCs were transfected with RAM siRNA 2 on 4 independent occasssions. After 48hrs RAM protein level was detected. c) 48hrs after transfection with three independent RAM siRNAs cell number was counted. d) Cells were infected with lentiviruses expressing RAM shRNA or control. After 7 days protein levels were detected by western blot. f) Colonies were stained with alkaline phosphatase. Colonies representative of those scored in figure 2d.

Figure S5

Ensembl Gene ID	Gene Name	log FC t'script	P value t'script	control polysome loading	Reference
			Pluripotency		
ENSMUSG00000024406	Pou5f1 (Oct4)	-0.188	1.43E-04	1.585	(Chen et al., 2008; Dunn et al., 2014; Kim et al., 2008)
ENSMUSG00000074637	Sox2	-0.092	5.72E-02	1.024	(Boroviak et al., 2015; Chen et al., 2008; Dunn et al., 2014; Kim et al., 200
ENSMUSG00000012396	Nanog	-0.291	3.78E-07	1.619	(Boroviak et al., 2015; Chen et al., 2008; Dunn et al., 2014; Kim et al., 200
ENSMUSG00000003032	Klf4	-0.140	5.35E-03	1.052	(Boroviak et al., 2015; Chen et al., 2008; Dunn et al., 2014; Kim et al., 200
ENSMUSG00000022346	Myc	0.092	2.60E-01	1.184	(Chen et al., 2008; Kim et al., 2008)
ENSMUSG00000037169	Mycn	-0.007	8.94E-01	1.643	(Chen et al., 2008)
ENSMUSG00000004040	Stat3	-0.056	2.66E-01	0.582	(Boroviak et al., 2015; Chen et al., 2008; Dunn et al., 2014)
ENSMUSG00000031681	Smad1	-0.155	2.91E-02	1.320	(Chen et al., 2008)
ENSMUSG00000005698	Ctcf	-0.0865	8.22E-02	0.860	(Chen et al., 2008)
ENSMUSG00000025056	Nr0b1	-0.081	1.10E-01	1.940	(Boroviak et al., 2015; Dunn et al., 2014; Kim et al., 2008)
ENSMUSG00000027490	E2f1	-0.164	1.35E-02	0.158	(Chen et al., 2008)
ENSMUSG00000021255	Esrrb	-0.248	3.53E-06	0.536	(Chen et al., 2008; Dunn et al., 2014)
ENSMUSG00000001910	Nacc1	-0.158	1.38E-03	0.699	(Chen et al., 2008)
ENSMUSG00000055024	Ep300	-0.104	5.44E-02	0.655	(Chen et al., 2008)
ENSMUSG00000051176	Zfp42	-0.111	2.04E-02	1.805	(Kim et al., 2008)
ENSMUSG00000017548	Suz12	-0.055	2.52E-01	0.801	(Chen et al., 2008)
ENSMUSG00000026380	Tfcp2l1	-0.246	9.94E-05	0.264	(Boroviak et al., 2015; Chen et al., 2008; Dunn et al., 2014)
ENSMUSG00000041483	Zfp281	-0.020	6.87E-01	0.540	(Kim et al., 2008)
ENSMUSG00000079509	Zfx	-0.207	5.03E-04	0.694	(Chen et al., 2008)
ENSMUSG00000055148	Klf2	0.046	5.23E-01	2.079	(Boroviak et al., 2015; Dunn et al., 2014)
ENSMUSG00000018604	Tbx3	-0.338	7.38E-08	0.747	(Dunn et al., 2014)
ENSMUSG00000034486	Gbx2	-0.082	1.98E-01	1.438	(Dunn et al., 2014)
ENSMUSG00000027547	Sall4	0.028	5.58E-01	0.971	(Dunn et al., 2014)
ENSMUSG00000020167	Tcf3	-0.079	1.26E-01	1.114	(Boroviak et al., 2015; Dunn et al., 2014)
ENSMUSG00000005148	Klf5	-0.060	2.23E-01	1.141	(Boroviak et al., 2015; Dunn et al., 2014)
ENSMUSG00000035478	Mbd3	-0.042	4.70E-01	1.170	(Dunn et al., 2014)
ENSMUSG00000051176	Zfp42	-0.111	2.04E-02	1.805	(Boroviak et al., 2015; Dunn et al., 2014)
ENSMUSG00000030353	Tead4	-0.125	7.10E-02	0.394	(Boroviak et al., 2015)
ENSMUSG00000026398	Nr5a2	-0.197	4.16E-04	0.854	(Boroviak et al., 2015)
ENSMUSG00000072419	Dppa2	0.111	3.08E-02	1.735	(Boroviak et al., 2015)
ENSMUSG00000020594	Pum2	-0.115	1.76E-02	0.854	(Boroviak et al., 2015)
ENSMUSG00000032446	Eomes	-0.317	1.24E-03	1.430	(Boroviak et al., 2015)
ENSMUSG00000042414	Prdm14	-0.517	3.93E-06	0.926	(Boroviak et al., 2015)
ENSMUSG00000032494	Tdgf1	-0.183	8.17E-04	1.506	(Boroviak et al., 2015)
ENSMUSG00000021835	Bmp4	-0.130	3.05E-02	1.039	(Boroviak et al., 2015)
ENSMUSG00000030117	Gdf3	-0.018	7.01E-01	2.476	(Boroviak et al., 2015)
ENSMUSG00000000730	Dnmt3l	0.558	3.07E-12	1.887	(Boroviak et al., 2015)
ENSMUSG00000025856	Pdgfa	-0.128	2.93E-02	0.667	(Boroviak et al., 2015)
ENSMUSG00000055799	Tcf7l1	-0.009	9.29E-01	1.630	(Boroviak et al., 2015)
ENSMUSG00000047751	Utf1	-0.213	9.06E-05	2.130	(Boroviak et al., 2015)
ENSMUSG00000067261	Foxd3	-0.277	7.35E-05	1.655	(Boroviak et al., 2015)
ENSMUSG00000067860	Zic3	-0.296	1.77E-05	1.019	(Boroviak et al., 2015)
ENSMUSG00000023902	Zscan10	-0.0085	8.69E-01	0.688	(Boroviak et al., 2015)
ENSMUSG00000050917	Fgf4	-0.419	4.24E-10	0.763	(Boroviak et al., 2015)
ENSMUSG00000090125	Pou3f1	-0.466	1.48E-03	0.725	(Boroviak et al., 2015)
ENSMUSG00000021848	Otx2	-0.238	1.72E-02	0.856	(Boroviak et al., 2015)
			Chromating remodelling		
ENSMUSG00000030034	Ino80b	-0.61	1.08E-06	2.13	(Wang et al., 2014)
ENSMUSG00000047989	Ino80c	-0.26	1.67E-04	0.66	(Wang et al., 2014)
ENSMUSG00000030689	Ino80e	-0.13	1.50E-02	1.02	(Wang et al., 2014)
ENSMUSG00000040865	Ino80d	-0.14	1.05E-02	0.14	(Wang et al., 2014)
ENSMUSG00000030689	Ino80e	-0.13	1.50E-02	1.02	(Wang et al., 2014)
			RNA PolII related		
ENSMUSG00000042487	Leo1	-0.01	9.01E-01	1.65	(Ponnusamy et al., 2009)
ENSMUSG00000045106	Ccdc73	0.07	4.97E-01	0.88	(Ponnusamy et al., 2009)
ENSMUSG00000005609	Ctr9	0.04	4.55E-01	0.60	(Ponnusamy et al., 2009)
ENSMUSG00000027304	Rtf1	-0.18	4.71E-03	1.20	(Ponnusamy et al., 2009)
ENSMUSG00000003437	Paf1	0.19	2.52E-04	1.05	(Ponnusamy et al., 2009)
ENSMUSG00000043866	Taf10	0.01	8.86E-01	0.73	(Pijnappel et al., 2013)
ENSMUSG00000024218	Taf11	-0.02	8.06E-01	1.13	(Pijnappel et al., 2013)
ENSMUSG00000048100	Taf13	-0.04	6.05E-01	0.54	(Pijnappel et al., 2013)
ENSMUSG00000025049	Taf5	-0.24	3.87E-03	1.38	(Pijnappel et al., 2013)
ENSMUSG00000028899	Taf12	-0.20	5.88E-03	0.84	(Pijnappel et al., 2013)
ENSMUSG00000078941	Ak6	-0.27	3.50E-03	1.12	(Pijnappel et al., 2013)

Figure S5 (related to Figure 3) List of pluripotency-associated factors
Transcript logFC in siRAM/Ct and polysome loading in ESCs of genes previously implicated in embryonic stem cell function. Median polysome loading is 0.99. For full references see main text.

Figure S6

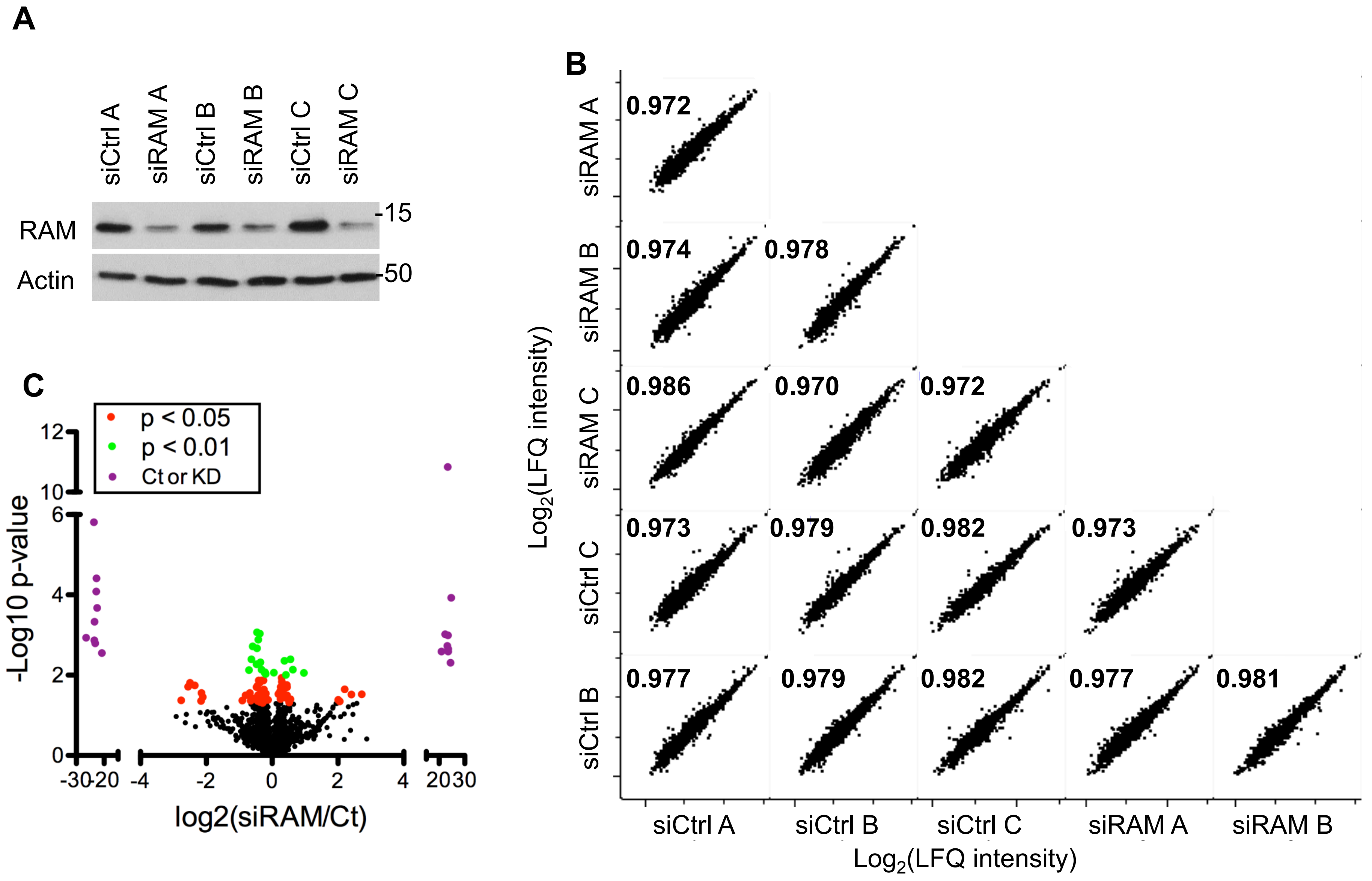

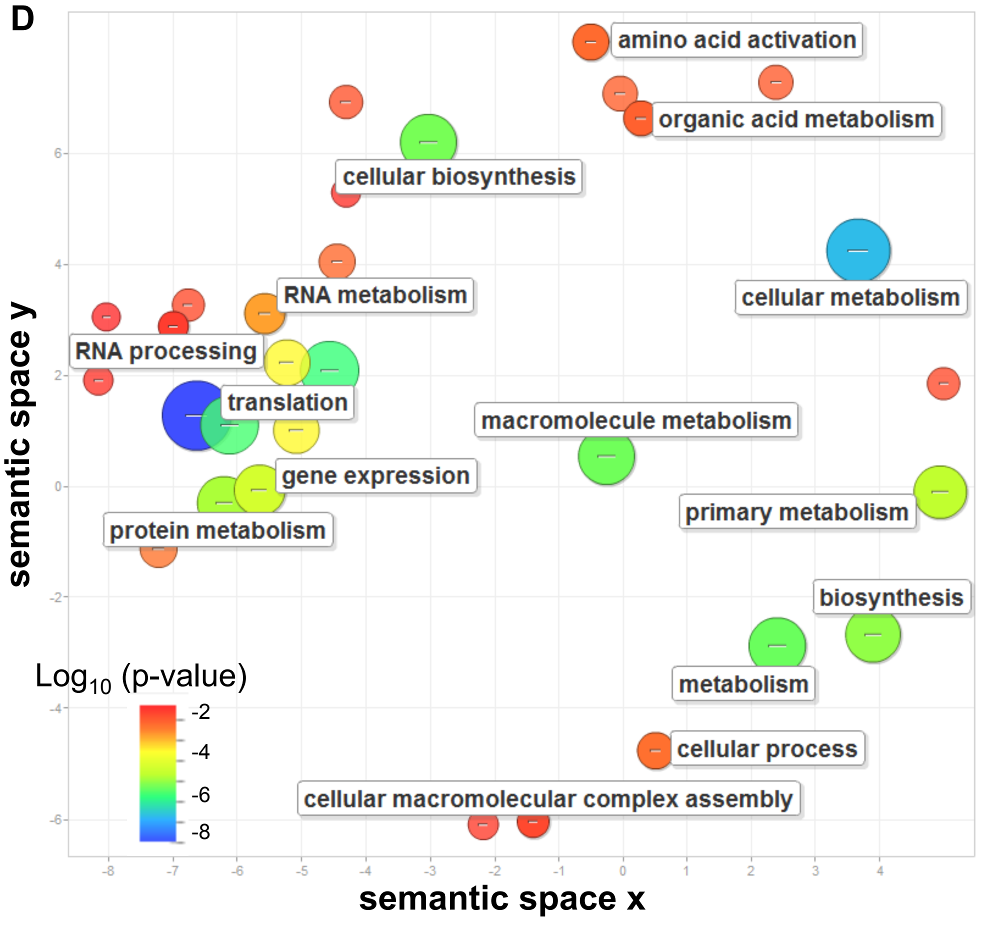

Figure S6 (related to Figure 3) LC-MS/MS analysis of the global proteome in response to RAM repression

ESCs were transfected with RAM siRNA pool or non-targeting control for 48hrs. a)Western blot analysis performed to detect proteins indicated. b) Samples analysed by LC-MS/MS, proteins identified and label –free quantified (LFQ) by MaxQuant (http://141.61.102.17/maxquant_doku/) and a scatter plot matrix for all biological replicates generated using Perseus framework (http://141.61.102.17/perseus_doku). Numbers indicate Pearson correlation between indicated samples. c) Cellular proteins quantified using LC-MS/MS. Each spot represents one of 2627 proteins analysed for differential expression. The y-axis plots the P-value (-log10 transformed) derived from Student's T-test across three biological replicates. The x-axis plots the mean log2 ratio of expression in RAM knock-down samples to control. The utmost left- and right-hand site spots (purple) represent proteins detected only in control or RAM knockdown samples respectively. d) The list of downregulated proteins after RAM knock-down (p value ≤ 0.05) analysed for the enrichment of GO biological process terms using DAVID database. Results rationalised using REVIGO. The colour and size of each bubble represent enrichment p-value (EASE score). The proximity of bubbles reflects related GO terms.

Figure S7

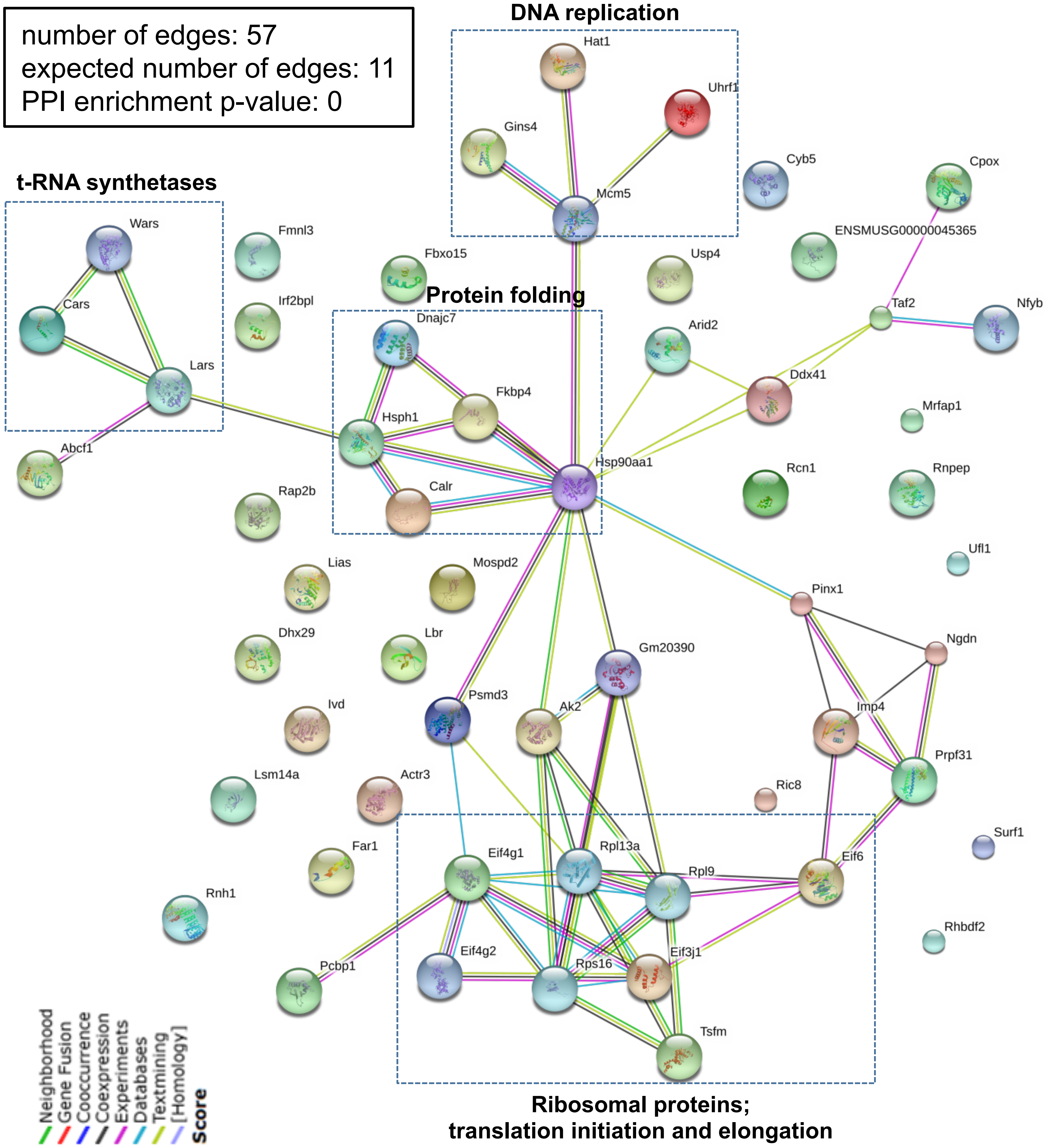

Figure S7 (related to Figure 3) STRING analysis of proteins downregulated after RAM knockdown
The list of downregulated proteins after RAM knockdown (p value ≤ 0.05) was analysed using STRING: functional protein association networks database (http://string-db.org/). Proteins were clustered based on the STRING Evidence Score. Gene clusters of interest are indicated.

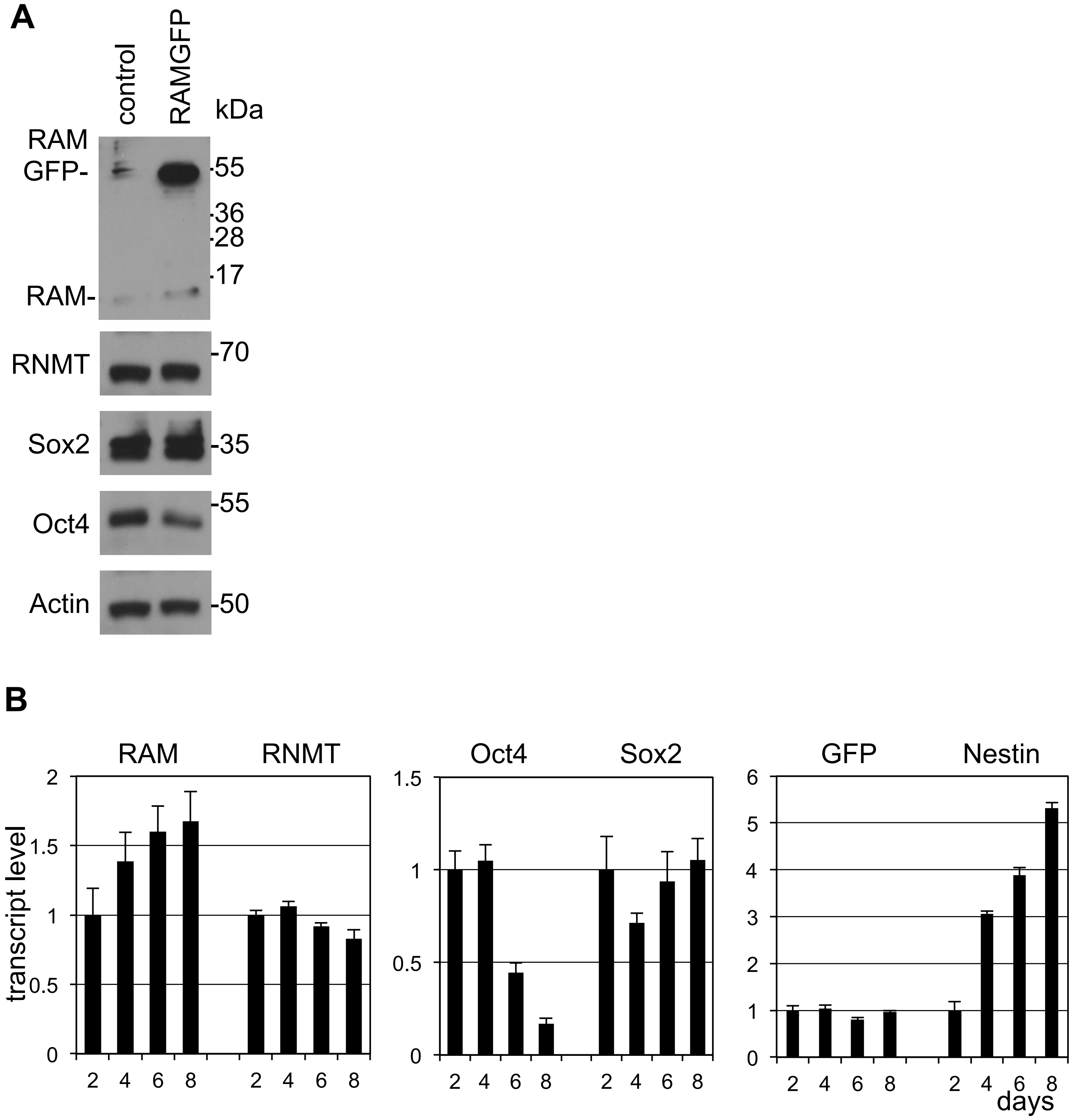

Figure S8 (related to Figure 4) Differentiation of ESC: RAM-GFP

a) In ESC:RAM-GFP and ESC:control, expression of the proteins indicated was detected by Western blot. b) ESCs stably expressing RAM-GFP were cultured according to a 9-day neural differentiation protocol. RT-PCR was performed for the genes indicated, expressed relative to Actin transcripts.

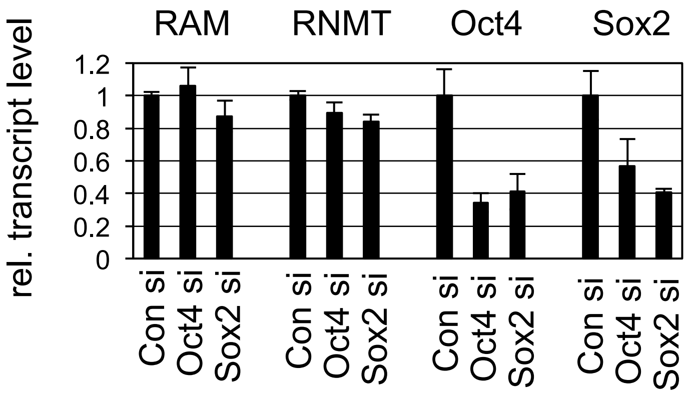

Figure S9 (related to Figure 5) RNMT and RAM transcript levels are not reduced by repression of Oct4 or Sox2
Expression of Oct4 and Sox2 was inhibited by transfection of siRNA pools for 72hrs. RTPCR performed to detect transcript expression relative to Actin transcripts.

Figure S10

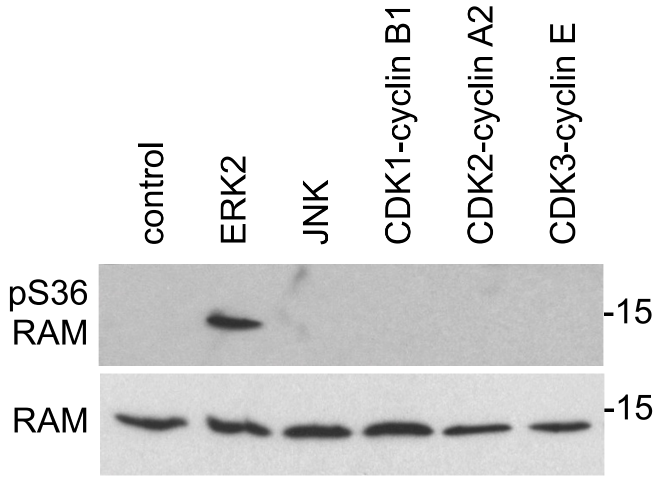

Figure S10 (related to Figure 6) ERK2 phosphorylates RAM
10ng GST-RAM subject to kinase reaction with kinases indicated was analysed by WB for pS36 RAM and total RAM.

Supplementary experimental procedures

ShRNA usage

For shRNA stable transduction non-targeting or RAM shRNA coding plasmids (MISSION Sigma) were co-transfected with envelope and packaging plasmids into HEK293T cells. The supernatant containing lentivirus was used to transduce mESCs and cells were selected with 1μg/ml Puromycin.

Alkaline Phosphatase

For clonal analysis, 200-600 cells were plated in the well of a 6-well plate and after 7 days fixed in citrate-acetone-formaldehyde and stained using the Alkaline Phosphatase (AP) kit (Sigma – Aldrich) according to the manufacturer's instructions. Colonies were scored for each experimental well as AP positive (cells all AP positive), mixed (containing cells both AP positive and negative) and or negative (cells all negative) (Figure S4E)

Polysome profile

Cells were incubated in 100 μg/ml cycloheximide for 10 min, washed in ice cold PBS supplemented with 100 μg/ml cycloheximide and extracts were collected in polysome lysis buffer: 15 mM Tris (pH7.5), 15 mM MgCl2, 0.3 M NaCl, 1 mM DTT, 1% Triton X-100, 100 mg/ml cycloheximide, 100 U/ml RNasin. 10% of extracts was kept as input and the rest layered onto 10 ml of 10%–50% sucrose steps and centrifuged at 18 000 x g for 2 hr at 4ºC. 0.5 ml fractions were collected and OD_{254} nm monitored. RNA was extracted from fractions using RNeasy Mini Kit (QIAGEN) according to manufacturer's instructions.

RTPCR

RNA was extracted using Trizol Reagent (Invitrogen). cDNA was synthesised using iScript cDNA Synthesis Kit (Bio-Rad). qRT-PCR was performed using QuantaBiosciences SYBR Green FastMix for iQ.

m7G IP

dT-purified RNA was IPed with 10 μl anti-7-methyl guanosine (Cole and Cowling, 2009). Transcripts were analyzed by real-time PCR and IPs expressed relative to inputs.

RNA-sequencing and analysis

Fractions 17-22, assessed to be polysomal, were purified by Phenol:Chloroform:Isoamyl Alcohol (25:24:1) (Ambion), pooled, and RNA precipitated with 2M of LiCl (Sigma). RNA from inputs was purified using Trizol Reagent (Invitrogen) according to manufacturer's instructions. RNA was sequenced at the Tayside Centre for Genomic Analysis. RNA was quality controlled using TapeStation (Agilent Technologies). RNA sequencing libraries were prepared with the TruSeq Stranded Total RNA with Ribo-Zero kit (Illumina). Sequencing was performed using NextSeq Series High Output Kit 2 × 75 bp (Illumina).

Reads were quality controlled using FastQC, mapped to (GRCm38/mm10) assembly of the mouse genome using STAR 2.5.1b (Dobin et al., 2013) and number of reads per gene quantified using htseq-count tool (Anders et al., 2015). Differentially expressed genes were identified using edgeR package (Robinson et al., 2010). Genes that had at least 1 counts per million (CPM) in at least 10 samples (inputs or polysomes) were analysed for differential expression. Polysome loading was calculated as ratio of gene abundance in polysome fraction over input in control sample. Median polysome loading 0.99.

Gene set tests were performed using ROAST which calculates the direction of changes of a gene set taking into account the magnitude of expression changes in the experiment (Wu et al., 2010). Gene sets tested were: pluripotency-associated factors (for full list see Fig S6), genes belonging to ESC expression group (upregulated in ESCs) or neurogenic progenitors (tNPs and nNPs) expression group (upregulated during N2B27 neural differentiation)(Abranches et al., 2009) (Table S1), the PluriNet protein-protein network shared by the pluripotent cells (Muller et al., 2008)(downloaded from Molecular Signatures Database, MSigDB http://software.broadinstitute.org/gsea/msigdb), genes upregulated in 6 human embryonic stem cell lines (Bhattacharya et al., 2004)(from MSigDB), genes coordinately upregulated in a compendium of mouse ESCs, which are shared with the human ESC-like module (Wong et al., 2008)(from MSigDB), genes involved in Neuronal System from Reactome Pathway Database (http://www.reactome.org)(from MSigDB), neuron markers - genes enriched in neurons in the adult mouse brain identified through correlation-based searches seeded with neuron cell-type specific gene expression patterns (Lein et al., 2007)(from MSigDB).

Proteomics

Cells were lysed in 8 M Urea, 100 mM Tris, pH 8.0 lysis buffer supplemented with 2x Complete protease inhibitor cocktail tablets (Roche) and 1x PhosSTOP phosphatase inhibitor cocktail tablets (Roche). 300 μg protein/sample was reduced with 10 mM DTT (Formedium) for 30 min

and thiol groups were alkylated with 50 mM iodoacetamide for 45 min. Samples were diluted to 0.8 M Urea and proteins digested with Trypsin Gold (Promega) two times. Samples were desalted on SepPak C18 SPE cartridges (Waters), washing with 0.1% trifluoroacetic acid (TFA) (Thermo), and eluted with 50% Acetonitrile (ACN) (VWR)/0.1%TFA. Eluted samples were dried, resuspended in 10 mM borate buffer, pH 9.3, 20% ACN and separated into 10 fractions on Dionex IonPac AS24 Hydroxide-Selective Anion-Exchange Column. Fractions were desalted as previously on Sep Pak tC18 elution plate, dried and resuspended in of 5% formic acid (Sigma). Peptides (0.7 μg/fraction) were analysed using Ultimate 3000 RSLCnano system (Thermo Scientific) coupled to a Q Exactive HF OrbiTrap Mass Spectrometer (Thermo Scientific). The tandem mass spectrometry analysis was carried out using HCD fragmentation. The data dependent acquisition mode was used to acquire MS/MS spectra from the top 20 most abundant ions from the full scan MS. The RAW data was processed using MaxQuant software (version 1.5.0.0) (Cox and Mann, 2008) using Label-free quantification (LFQ) mode. MS/MS spectra were searched against mouse UniProt database (17/03/15) using the Andromeda search engine. Peptide and protein level identification were both set to maximum false discovery rate of 1%. For protein quantification match between runs function was enabled and minimum ratio count was set to two. Protein groups identified as contaminants or reverse sequences were removed. Statistical analysis was done in Microsoft Excel. P values were calculated using *Student's t test.* For calculation of log2 ratio in RAM knock-down samples to control values 0 were replaced with 1. The Gene Ontology (GO) analysis was performed using DAVID Functional Annotation Tool (Huang da et al., 2009). Significantly enriched GO biological process terms (*modified Fisher Exact P-Value* – EASE score ≤ *0.05)* were visualised using REVIGO (Supek et al., 2011). GO term enrichment analysis was performed using STRINGdb Bioconductor package (Franceschini et al., 2013).

Western Blotting

Cells were lysed in lysis buffer (10 mM Tris (pH 7.05), 50 mM NaCl, 50 mM NaF, 10% glycerol, 0.5% Triton X-100 and protease inhibitors). Cell extracts were normalised for protein content and typically 8-20 ug was analysed by Western Blot. Primary antibodies used were raised against RNMT (polyclonal sheep), RAM (polyclonal sheep), pS36 RAM (polyclonal sheep), ERK 1/2 (Cell Signalling 9102) pERK 1/2 (Cell Signalling 9101S), Oct4 (Abcam 18976), Sox2 (Cell Signaling 4900), Nanog (Abcam 80892), Klf4 (Santa Cruz 20691), Actin (Abcam 3280.) FG-M2 (Sigma F1804) and GFP (Roche 11814460001), Ubiquitin (Dako Z0458).

Immunofluorescence

ES cells were seeded onto cover glass previously coated with 0.1% gelatin whereas neurons were seeded onto cover glass coated with Laminin (Sigma) for 2 hr. Cells were fixed with PFA 4% for 30 min, permabilised with 1% Triton X-100 in PBS and then blocked with 1% Donkey serum in PBS with 0.05% Tween 20 for 30 min. Cells were then incubated for 1 hr with primary antibodies RNMT (1:500, in house), RAM (1:100, in house), Oct4 (1:500, Abcam) Pax 6 (1:500, Santa Cruz) and beta-III-tubulin (Sigma T5076, 1:1000). Cells were then washed three times with PBS to be then incubated for 1 hr with secondary antibodies (Alexa) in the dark. Nuclei were stained with DAPI (Sigma).

hES cells were washed twice with PBS, fixed for 10 min with 10% neutral buffered formalin (Sigma). Cells were then washed with PBS and treated with ice cold 90% methanol for 5 min at -20°C. Cells were then blocked for 1 hr with 4% BSA in PBS/0.1 % Triton X-100 (blocking buffer), then treated as above. Antibodies used: beta-III-tubulin (Sigma T5076, 1:1000) and Pax6 (Abcam 154253, 1:200).

High-resolution images were collected with an imaging system (DeltaVision Restoration; Applied Precision) using a 40X/ 1.514 oil or 60X/1.42 oil (Olympus) objective lens. Images were then processed using OMERO software.

Quantification of the fluorescence intensity of Oct4, Nanog and Sox2 in cells transfected with three independent siRNAs or a non-targeting control for 48 hours. Measurements were performed using the freeware image analysis software, Image J. Data indicate the mean ± standard deviation from at least ten images from two independent experiments.

Kinase assay

500ng GST-RAM was incubated with 100ng kinase at 37ºC for 30 minutes in 50mM Tris pH 7.5, 0.1% B-mercaptoethanol, 10mM Mg Acetate, 100µM ATP and 1µM Microcystin LR. Kinases used were ERK2 (DSTT), CDK2-cyclin A2 and JNK (DSTT), CDK1-cyclin B1 (NEB) and CDK3-cyclin E (Thermo Scientific). Kinases were verified as active by DSTT (Aregger et al., 2016)

RAM-GFP expression

RAM-GFP was cloned into the vector pPyPCAGIP (Chambers et al., 2003). In this vector RAM-GFP is driven by the CAG chimeric enhancer/promoter consisting of a CMV early enhancer, chicken beta-actin promoter, first exon and first intron and the splice acceptor from rabbit beta

globin (Niwa et al., 1991). RAM is expressed upstream of IRES-Puro allowing puromycin selection. This promoter is resistant to silencing in ESCs.

References

Abranches, E., Silva, M., Pradier, L., Schulz, H., Hummel, O., Henrique, D., and Bekman, E. (2009). Neural differentiation of embryonic stem cells in vitro: a road map to neurogenesis in the embryo. PloS one *4*, e6286.
Anders, S., Pyl, P.T., and Huber, W. (2015). HTSeq--a Python framework to work with high-throughput sequencing data. Bioinformatics (Oxford, England) *31*, 166-169.
Aregger, M., Kaskar, A., Varshney, D., Fernandez-Sanchez, M.E., Inesta-Vaquera, F.A., Weidlich, S., and Cowling, V.H. (2016). CDK1-Cyclin B1 Activates RNMT, Coordinating mRNA Cap Methylation with G1 Phase Transcription. Mol Cell *61*, 734-746.
Bhattacharya, B., Miura, T., Brandenberger, R., Mejido, J., Luo, Y., Yang, A.X., Joshi, B.H., Ginis, I., Thies, R.S., Amit, M.*, et al.* (2004). Gene expression in human embryonic stem cell lines: unique molecular signature. Blood *103*, 2956-2964.
Chambers, I., Colby, D., Robertson, M., Nichols, J., Lee, S., Tweedie, S., and Smith, A. (2003). Functional expression cloning of Nanog, a pluripotency sustaining factor in embryonic stem cells. Cell *113*, 643-655.
Cole, M.D., and Cowling, V.H. (2009). Specific regulation of mRNA cap methylation by the c-Myc and E2F1 transcription factors. Oncogene *28*, 1169-1175.
Cowling, V.H. (2010). Enhanced mRNA cap methylation increases cyclin D1 expression and promotes cell transformation. Oncogene *29*, 930-936.
Cox, J., and Mann, M. (2008). MaxQuant enables high peptide identification rates, individualized p.p.b.-range mass accuracies and proteome-wide protein quantification. Nature biotechnology *26*, 1367-1372.
Dobin, A., Davis, C.A., Schlesinger, F., Drenkow, J., Zaleski, C., Jha, S., Batut, P., Chaisson, M., and Gingeras, T.R. (2013). STAR: ultrafast universal RNA-seq aligner. Bioinformatics (Oxford, England) *29*, 15-21.
Franceschini, A., Szklarczyk, D., Frankild, S., Kuhn, M., Simonovic, M., Roth, A., Lin, J., Minguez, P., Bork, P., von Mering, C.*, et al.* (2013). STRING v9.1: protein-protein interaction networks, with increased coverage and integration. Nucleic Acids Res *41*, D808-815.
Huang da, W., Sherman, B.T., and Lempicki, R.A. (2009). Systematic and integrative analysis of large gene lists using DAVID bioinformatics resources. Nat Protoc *4*, 44-57.
Lein, E.S., Hawrylycz, M.J., Ao, N., Ayres, M., Bensinger, A., Bernard, A., Boe, A.F., Boguski, M.S., Brockway, K.S., Byrnes, E.J.*, et al.* (2007). Genome-wide atlas of gene expression in the adult mouse brain. Nature *445*, 168-176.
Muller, F.J., Laurent, L.C., Kostka, D., Ulitsky, I., Williams, R., Lu, C., Park, I.H., Rao, M.S., Shamir, R., Schwartz, P.H.*, et al.* (2008). Regulatory networks define phenotypic classes of human stem cell lines. Nature *455*, 401-405.
Niwa, H., Yamamura, K., and Miyazaki, J. (1991). Efficient selection for high-expression transfectants with a novel eukaryotic vector. Gene *108*, 193-199.
Robinson, M.D., McCarthy, D.J., and Smyth, G.K. (2010). edgeR: a Bioconductor package for differential expression analysis of digital gene expression data. Bioinformatics (Oxford, England) *26*, 139-140.
Supek, F., Bosnjak, M., Skunca, N., and Smuc, T. (2011). REVIGO summarizes and visualizes long lists of gene ontology terms. PloS one *6*, e21800.
Wong, D.J., Liu, H., Ridky, T.W., Cassarino, D., Segal, E., and Chang, H.Y. (2008). Module map of stem cell genes guides creation of epithelial cancer stem cells. Cell Stem Cell *2*, 333-344.

Wu, D., Lim, E., Vaillant, F., Asselin-Labat, M.L., Visvader, J.E., and Smyth, G.K. (2010). ROAST: rotation gene set tests for complex microarray experiments. Bioinformatics (Oxford, England) *26*, 2176-2182.
